# Neuroprotection by YZG-331 in postoperative cognitive dysfunction *via* Mg^2+^-dependent blockade of NMDA receptors

**DOI:** 10.1016/j.apsb.2026.07.025

**Published:** 2026-07-20

**Authors:** Han Guo, Jiaojiao Zhao, Keying Tian, Qinghui Guo, Siruan Chen, Yuhan Ge, Wenyan Guo, Xingchen Lin, Hui Bai, Lei Yang, Lei Yang, Xia Qin, Panpan Zhang, Zhanfeng Jia, Ruifang Zheng, Zuxiao Yang, Yun Stone Shi, Dezhi Kong, Tengfei Ji, Wei Zhang

**Affiliations:** a Department of Pharmacology of Chinese Materia Medica, Institution of Chinese Integrative Medicine, The Key Laboratory of Tranquilizing TCM, Hebei Provincial Administration of Traditional Chinese Medicine, The Key Laboratory of Neural and Vascular Biology, Ministry of Education, Research Unit of Digestive Tract Microecosystem Pharmacology and Toxicology, Chinese Academy of Medical Sciences, State Key Laboratory of New Pharmaceutical Preparations and Excipients, Hebei Medical University, Shijiazhuang 050017, China; b Department of Oral Medicine, The Second Hospital of Hebei Medical University, Shijiazhuang 050000, China; c Model Animal Research Center, Medical School, Nanjing University, Nanjing 210032, China; d Guangdong Institute of Intelligence Science and Technology, Zhuhai 519031, China; e Center for Biological Science and Technology, Advanced Institute of Natural Sciences, Beijing Normal University, Zhuhai 519087, China; f Department of Cardiac Ultrasound, The Second Hospital of Hebei Medical University, Shijiazhuang 050000, China; g School of Basic Medical Sciences, Capital Medical University, Beijing 100069, China; h Department of Pharmacology, Hebei Medical University, Shijiazhuang 050017, China; i Xinjiang Key Laboratory of Uygur Medical Research, Xinjiang Institute of Materia Medica, Urumqi 841100, China; j Institute of Materia Medica, Chinese Academy of Medical Sciences and Peking Union Medical College, Beijing 100050, China

**Keywords:** Postoperative cognitive dysfunction (POCD), Cognitive function, YZG-331, NMDA receptors, Mg^2+^, Neuroprotection

## Abstract

Postoperative cognitive dysfunction (POCD) is a debilitating neurological complication that occurs following surgery, for which no effective interventions are currently available. Here, we identified YZG-331, a novel adenosine analog, as a promising neuroprotective agent that rescues cognitive deficits and mitigates dendritic spine loss in a murine POCD model. Integrated proteomic profiling and bioinformatics pathway analysis revealed that YZG-331 selectively targets NMDA receptor (NMDAR) signaling. Electrophysiological recordings and living-cell calcium imaging demonstrated that YZG-331 suppressed NMDAR-mediated currents and calcium influx in a dose-dependent manner. Furthermore, Mg^2+^ is required for YZG-331 to inhibit NMDAR activity. YZG-331 disrupts the formation of NMDAR condensates in the presence of Mg^2+^, thereby attenuating NMDAR-mediated excitotoxicity. Our findings establish YZG-331 as a prospective therapeutic candidate for POCD and reveal Mg^2+^-dependent receptor modulation as a novel pharmacological strategy for targeting NMDAR-associated neuropathologies.

## Graphical abstract

Perioperative neurotoxicity drives NMDAR-dependent Ca^2+^ overload and synaptodendritic degeneration. YZG-331 enhances Mg^2+^ blockade of NMDARs, curbing Ca^2+^ influx to restore hippocampal synaptic integrity.

**Figure:**
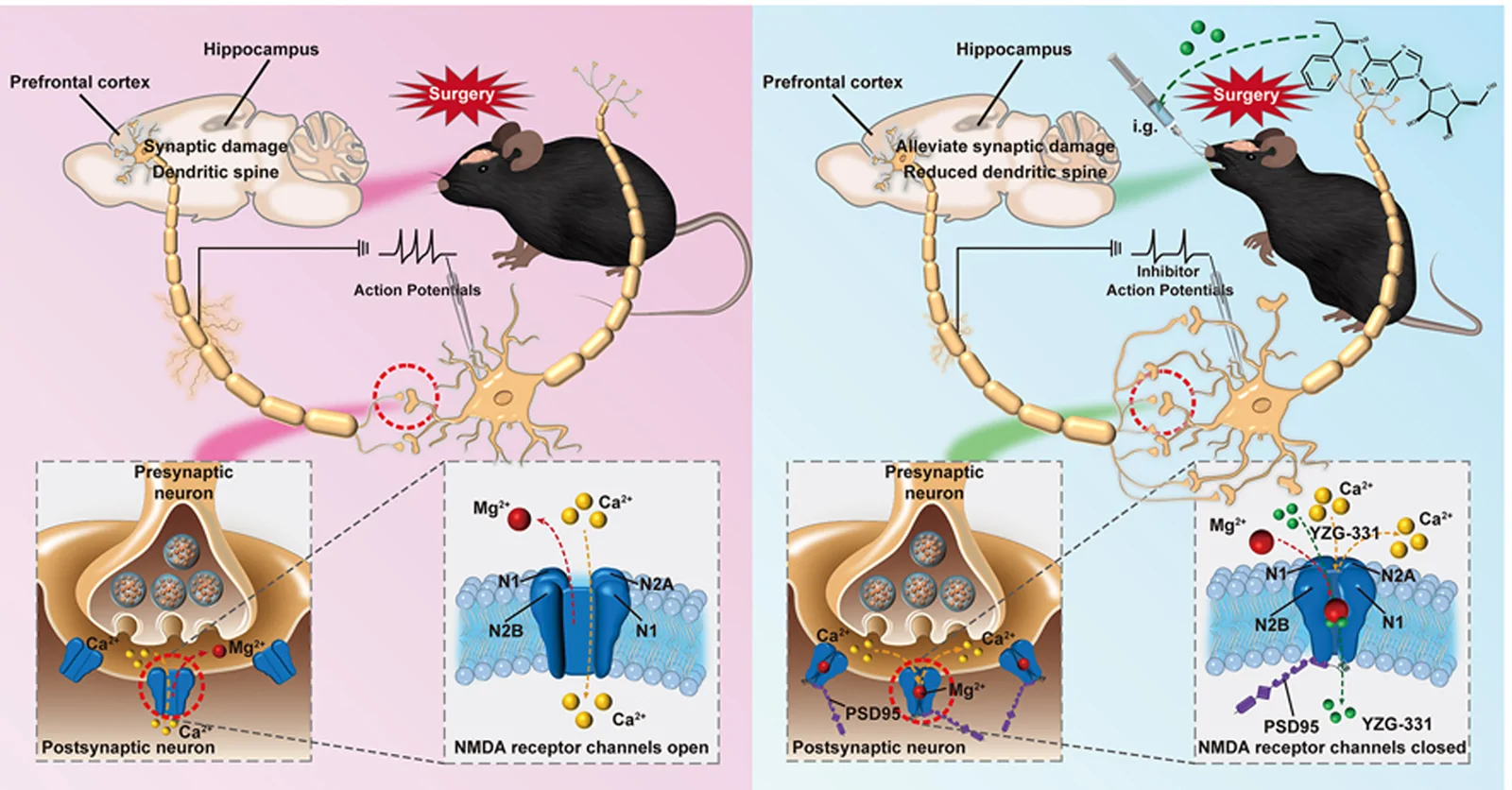

## 1 Introduction

Postoperative cognitive dysfunction (POCD) is a debilitating neurocognitive syndrome characterized by deficits in attention, memory, language, and social function. It represents a significant challenge within the field of perioperative medicine^1^, with documented cases affecting 40%–70% of patients undergoing surgeries, such as cardiac procedures or hip arthroplasty. The symptoms associated with POCD can persist from several days to months^2^, ^3^, ^4^, ^5^, ^6^, ^7^, ^8^ thereby delaying recovery and accelerating long-term cognitive decline. Additionally, POCD is associated with an increase in the 5-year mortality risk and imposes considerable socioeconomic burdens^9^. Despite its clinical severity, current management strategies are predominantly supportive, underscoring the urgent need to develop effective therapeutic interventions for this condition.

Despite posing a significant risk to patients, the underlying mechanisms of POCD remain under investigation. Evidence implicates multiple factors in the pathogenesis of POCD, including neuroinflammation, glutamate toxicity, synaptic impairment, and *N*-methyl-d-aspartate receptor (NMDAR) dysfunction^10^. NMDARs are critical mediators of synaptic plasticity and have dual effects on cognition. While NMDAR activation is essential for long-term potentiation (LTP) and memory consolidation^11^, ^12^, ^13^, ^14^, pathological overactivation of NMDAR triggers excessive Ca^2+^ influx. This Ca^2+^ dysregulation promotes mitochondrial dysfunction, oxidative stress, and synaptic loss^15^^,^^16^. Perioperative stressors further exacerbate this cascade^17^^,^^18^ by disrupting dendritic spine stability and impairing synaptic plasticity in key cognitive regions, such as the hippocampus and prefrontal cortex^19^, ^20^, ^21^. Consequently, NMDAR-dependent excitotoxicity is recognized as the central mechanism linking surgical insults to POCD. However, despite compelling evidence for this pathway, targeted pharmacological interventions remain underdeveloped.

YZG-331, a rationally designed *N*^6^-arylethyl adenosine derivative evolved from *Gastrodia elata*-derived *N*^6^-(4-hydroxyphenylmethyl) adenosine (NHBA), which was recently approved for clinical trials, demonstrates superior neuropharmacokinetic properties, including BBB penetrability and thalamocortical biodistribution selectivity^22^. Preclinical evaluations have revealed the multimodal mechanisms of action of YZG-331, as outlined below: 1) adenosine receptor subtype-biased agonist, 2) P-glycoprotein transport modulation enhancing CNS bioavailability, and 3) glutamate-glutamine cycle modulation *via* GAD65 (glutamic acid decarboxylase 65) upregulation^23^^,^^24^. Furthermore, quantitative microdialysis has demonstrated that YZG-331 reduces extracellular glutamate levels in the medial prefrontal cortex and ventromedial hypothalamus^25^^,^^26^. Given that previous studies have identified glutamate toxicity and disturbed glutamate metabolism as being involved in the pathological mechanism of POCD^27^, we hypothesized that YZG-331 has the potential to alleviate the effects of POCD.

Here, we elucidated the neuroprotective effects of YZG-331 using a murine POCD model. In this murine POCD model, YZG-331 rescued hippocampal-dependent spatial memory deficits and preserved dendritic spine density. Mechanistically, YZG-331 inhibits the NMDARs current in Mg^2+^-dependent manner, thereby reducing the synaptic NMDAR-dependent calcium influx and minimizing excitotoxic damage to neurons. These results thus identify YZG-331 as the first drug shown to mechanistically link Mg^2+^-dependent. NMDAR inhibition to the mitigation of excitotoxicity. Our findings establish YZG-331 as a novel NMDAR modulator with the potential for perioperative neuroprotection.

## 2 Materials and methods

### 2.1 Animals

Male C57BL/6J mice (8 weeks old; Charles River Labs) were maintained under controlled conditions (22 °C, 50% humidity, 12-h light cycle) with free access to food/water. After 7-day acclimation, the mice were randomized into control, surgical, or treatment groups. All protocols followed NIH guidelines (NIH Publication, 8th Edition, 2011) and were approved by the Ethics Committee of Hebei Medical University (IACU-HEBMU-2021020).

### 2.2 Pharmaceuticals

YZG-331 [*N*^6^-(4-hydroxybenzyl) adenosine riboside] was synthesized and authenticated by Prof. Jiangong Shi's laboratory (Institute of Materia Medica, CAMS & PUMC; Batch number 201110718). The treatment group mice received 10, 40, 80 mg/kg YZG-31 suspended in 0.5% carboxymethyl cellulose sodium (CMC-Na, Sigma, USA) *via* oral gavage 24 h preoperatively, while surgical controls received equivalent volumes of CMC-Na vehicle (≤200 μL/mouse)^28^.

### 2.3 Anesthesia and surgery

**Figure 1:**
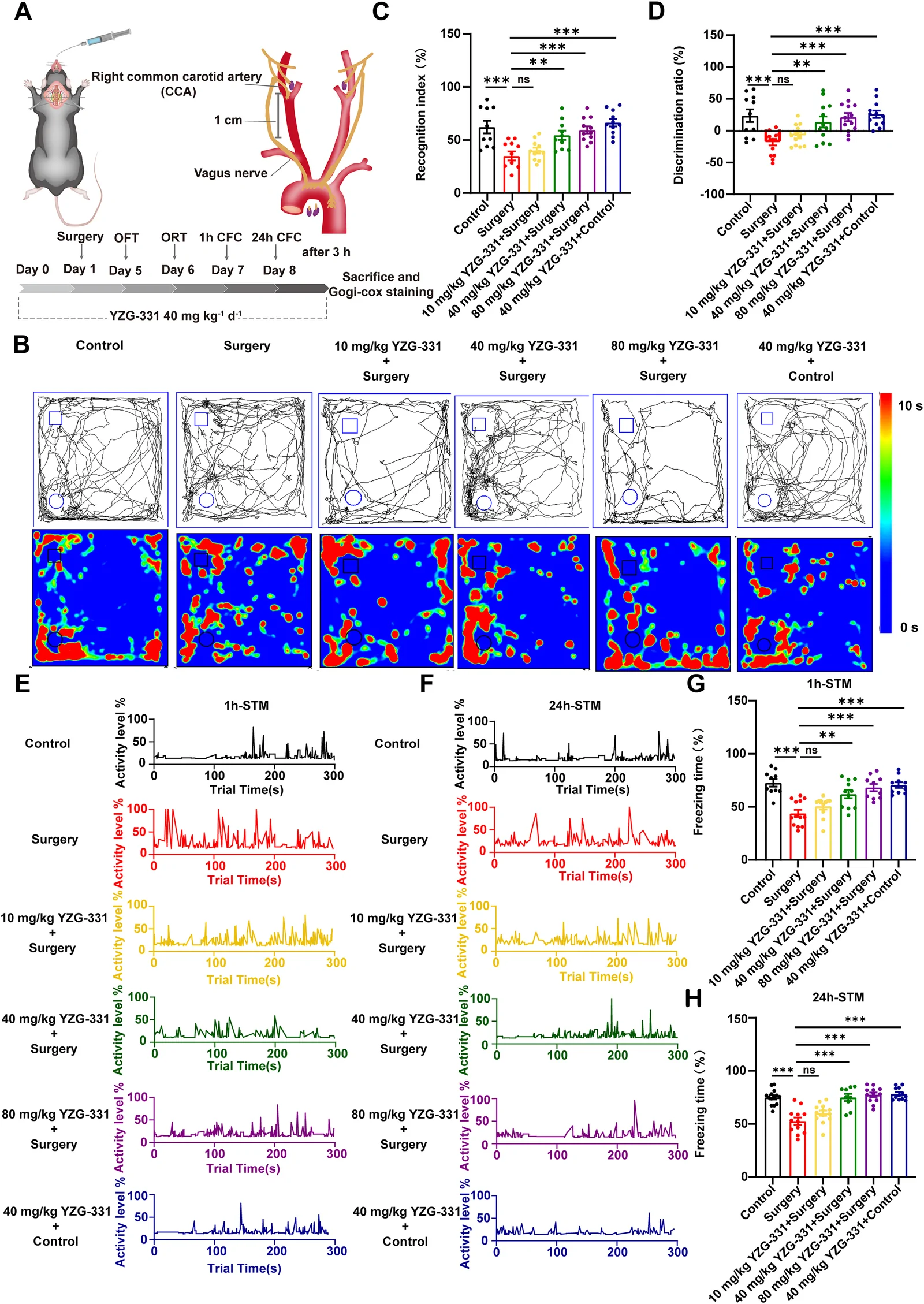
YZG-331 improved cognitive and memory deficits in anesthesia/surgery mice. (A) Schematic representation of experimental groups. (B) Representative movement tracks and time heatmap in the novel object recognition test. (C, D) Recognition and discrimination experiments of new object recognition in groups of mice (Control *n* = 10; Surgery *n* = 11; 10 mg/kg YZG-331+Surgery *n* = 11; 40 mg/kg YZG-331+Surgery *n* = 10; 80 mg/kg YZG-331+Surgery *n* = 11; 40 mg/kg YZG-331+Control *n* = 11). (E–H) Represent short-term memory (STM, 1 h, E, G), long-term memory (LTM, 24 h, F, H) and quantification of fear conditioning in six groups of mice (Control *n* = 10; Surgery *n* = 13; 10 mg/kg YZG-331+Surgery *n* = 11; 40 mg/kg YZG-331+Surgery *n* = 10; 80 mg/kg YZG-331+Surgery *n* = 11; 40 mg/kg YZG-331+Control *n* = 11). The threshold for the freezing state was set at 10% according to the actual condition of the mice. All data were analyzed using one-way ANOVA with Dunnett's test and are presented as mean ± SEM. *∗P* < 0.05; *∗∗P* < 0.01; ∗*∗∗P* < 0.001; ns, no significance.

Right carotid artery exposure surgery was used to induce POCD, as previously described^29^^,^^30^. Postoperative cognitive dysfunction was modeled *via* right common carotid artery exposure under 3% sevoflurane anesthesia (30% O_2_/70% N_2_ carrier gas), with continuous respiratory monitoring using Datex Capnomac™ (Aeonmed VP 300, China). The core temperature was maintained at 37.0 ± 0.5 °C using a feedback-regulated heating pad. Following ≥30 min of anesthetic stabilization, a 1 cm midline cervical incision exposed the right common carotid artery, which was microsurgically isolated from the vagal trunk (3.5–5 mm segment) without neurovascular compromise. The operative field was irrigated with sterile saline and closed with 6-0 vicryl sutures under aseptic conditions (15 min total procedure time) (Fig. 1A). Postoperative analgesia included subcutaneous bupivacaine (3 mg/kg), with animals recovering spontaneously after 2 h of anesthesia.

### 2.4 Open field test

The open-field test was conducted in a 45 cm × 45 cm × 45 cm arena with defined zones (10 cm peripheral border *vs.* central area), where mice underwent 5 min behavioral recording following central zone initiation, utilizing the SMART® tracking system (Panlab, Spain) to quantify total ambulatory distance and peripheral thigmotaxis.

### 2.5 Object recognition task

Object recognition task was conducted to assess non-spatial memory consolidation in rodents according to established methodologies^31^. The mice underwent a 5 min acclimatization period in an open field for three days. During the training trials, two identical ethanol-sanitized (75%) wooden cubes were positioned 10 cm from the arena walls. The animals were oriented away from the objects for 1 min of habituation, followed by 5 min of unrestricted exploration. In the test phase (1 h post-training), one object was replaced with a novel geometric counterpart of identical material and dimensions. Exploratory behavior (sniffing, whisker contact ≤2 cm) was quantified during a 5 min session using SMART® tracking software, excluding non-cognitive interactions (*e.g.*, resting and passive observation). Memory performance was calculated using Eq. (1):

(1) Noveltypreferenceratio(NOR)=Timeornumberofnewobjects/(Timeornumberofnewobjects+Timeornumberofoldobjects)

The discrimination index was calculated using Eq. (2):

(2) Discriminationindex(DI)=(Timeornumberofnewobjects–Timeornumberofoldobjects)/(Timeornumberofnewobjects+Timeornumberofoldobjects)

### 2.6 Contextual fear conditioning

Rodent fear memory was assessed using a computer-controlled conditioning system (Panlab, Barcelona, Spain) with a standardized protocol^31^. Mice underwent 3-day acclimatization (5 min/day) followed by training: 120 s baseline observation preceding three 2 s footshocks (0.7 mA) delivered through stainless steel grid floorings. Memory retention was tested at 1 h (short-term memory) and 24 h (long-term memory) post-conditioning *via* 5 min of context re-exposure without aversive stimuli. Freezing behavior defined as motionless crouching posture with piloerection and defecation, excluding respiratory movements or tremors was quantified as Eq. (3):

(3) Freezing(%)=(Immobilityduration/300)×100

### 2.7 Golgi-Cox staining

Brains were harvested 8 days post-surgery for Golgi-Cox staining^32^ using a commercial Kit (#PK401, FD Neuro Technologies, Columbia, MD, USA). Mice underwent terminal anesthesia *via* 3% sevoflurane in 30% O_2_/70% N_2_, followed by CO_2_ asphyxiation in a sealed euthanasia chamber with continuous physiological monitoring of the parameters. The brains were immediately extracted and immersed in a pre-mixed impregnation solution (A + B) for 21 days at 22–25 °C under light-protected conditions. The tissue was subsequently cryoprotected in solution C (7 days), coronally sectioned at 100 μm using an oscillating tissue slicer, and mounted on gelatin-coated slides. The sections underwent sequential ethanol dehydration and xylene clearing prior to coverslipping. Hippocampal CA1 and cortical pyramidal neurons were imaged using a Pannoramic scanner scanning software (CaseViewer2.4 3DHISTECH). Dendritic arbors were reconstructed using NeuronJ (ImageJ) for quantitative analysis: 1) Sholl analysis: Dendritic complexity was quantified *via* concentric circle intersections (20 μm radial increments). 2) Spine density: secondary dendrites were analyzed from primary branch origins by blinded observers.

### 2.8 Cell culture

Primary cortical neuron cultures were maintained as previously described^33^. Fetuses were removed by cesarean section from the mice fetuses on gestational days. The cerebral cortex was isolated from fetal mice brains and minced, followed by digestion with 0.125% trypsin. The neurons were cultured in neurobasal medium containing 2% B-27 and 0.2 mol/L GlutaMAX™ (Thermo Scientific). Half the volume of the medium was replaced every 3–4 days. HEK293T cells were cultured in 90% high-glucose Dulbecco's modified Eagle's medium (DMEM Thermo Scientific) with 10% fetal bovine serum (FBS Thermo Scientific). The cells were incubated at 37 °C in 5% CO_2_.

### 2.9 Scanning electron microscope

The culture medium was aspirated from primary neurons cultured on coverslips. Within 3 min, the cells were rinsed with PBS (pH 7.4) to remove contaminants. Primary fixation was performed using 2% glutaraldehyde/4% paraformaldehyde in 0.1 mol/L phosphate buffer (PB, pH 7.4) for 2 h at room temperature (RT), followed by three PB washes (15 min each). Secondary fixation was performed using 1% osmium tetroxide (OsO_4_) in PB for 1.5 h (RT, light-protected), followed by PB rinsing. Ethanol dehydration was performed using a graded series (30%–100%, 15 min/step) before transitioning to isoamyl acetate. Critical point drying (CPD) using liquid CO_2_ was performed before mounting on carbon tape-adhered SEM stubs. The specimens were sputter-coated with a 15 nm Au/Pd layer (30 s, 20 mA), achieving surface conductivity (<10 Ω/cm^2^ resistance) prior to SEM imaging.

### 2.10 Transmission electron microscopy

The culture medium from well-cultured cells (at ≤ 70% confluency) was aspirated. The cells were then immediately quenched with pre-chilled electron microscopy fixative (4 °C) and fixed at room temperature in the dark for 5 min. Following fixation, the cells were gently detached by scraping in a single direction, taking care to prevent rupture. Primary fixation was followed by three 0.1 mol/L phosphate buffer (PB, pH 7.0) rinses (15 min/wash) and secondary fixation in 1% OsO_4_/PB (pH 7.4) for 2 h (RT, light-protected), with three subsequent PB rinses (pH 7.4, 15 min/wash). Dehydration was performed using a graded ethanol series (30%, 50%, 70%, 80%, 95%, and 100% *v*/*v*), with each step lasting 20 min, followed by two rinses in absolute acetone (100%, 15 min each) prior to epoxy resin infiltration.

### 2.11 Microvia multi-array electrode technology

The MEA chips were sterilized using ultraviolet light for 20 min and coated with 0.1 mg/mL poly-d-lysine for 8 h. Cells were planted on MEA chips at a density of 1000 cells/mm^2^ and incubated at 37 °C with 5% CO_2_ every three days with a half-volume fluid exchange. During the formal experiments, the electrical activity of the neural network was continuously monitored and recorded using a Maestro Pro recording system at 37 °C and 5% CO_2_. The data were recorded using a BioCore V4 (Axion BioSystems, USA) with a data acquisition frequency set at 20 KHz and analyzed using the AxIS Navigator (Axion BioSystems, USA).

### 2.12 Proteomics analysis

Neurons cultured *in vitro* were lysed in urea buffer (8 mol/L urea, 100 mmol/L Tris–HCl, pH 8.0) containing protease inhibitors and ultrasonicated. Protein concentration was determined using the BCA assay. For each 100 μg protein aliquot, ice-cold acetone (4 × volume) was added for precipitation at −40 °C (4 h). Precipitated proteins were reduced/alkylated prior to digestion with trypsin (1:50 *w*/*w* enzyme-to-substrate ratio) in 50 μL triethylammonium bicarbonate buffer at 37 °C overnight. The samples were then analyzed as described previously^34^.

The tryptic peptides were desalted, lyophilized, and labeled using a TMT 6-plex Kit (Thermo Fisher Scientific, 90,061) according to the manufacturer's protocol. Labeled peptides were pooled, desalted, and fractionated into 11 fractions *via* high-pH reverse-phase chromatography using a BEH C18 column (Waters). Fractions were reconstituted in 0.1% formic acid and analyzed by nanoLC-Orbitrap Fusion Lumos Tribrid MS operated in data-dependent MS2 acquisition (DDA) mode. MS raw files were processed using Proteome Discoverer 2.1 with Sequest HT against the UniProt database (https://www.uniprot.org/). Differentially expressed proteins were identified using volcano plot analysis (R package, ggplot2). Functional annotation was performed using DAVID v6.8 (https://david.ncifcrf.gov) for Gene Ontology (GO) enrichment and pathway analysis. Heatmaps were generated to visualize the protein expression patterns.

### 2.13 Brain slices preparation and electrophysiological recording

Fourteen-day-old male C57BL/6 mice (6–7 g) were anesthetized with sevoflurane and decapitated. The brain was rapidly dissected and immersed in oxygenated ice-cold slicing solution (in mmol/L: 210 Sucrose, 25 NaHCO_3_, 10 Glucose, 1.25 NaH_2_PO_4_, 25 NaHCO_3_, 2.5 KCl, 7 MgCl_2_, and 0.5 CaCl_2_; pH 7.4, adjusted with HCl) as described previously^35^. Coronal hippocampal slices (300 μm) were prepared with a vibratome (VT1200S; Leica, Germany), incubated in oxygenated artificial cerebrospinal fluid (ACSF in mmol/L: 119 NaCl, 1 NaH_2_PO_4_, 11 Glucose, 2.5 KCl, 2.5 CaCl_2_, 1.3 MgSO_4_, 26 NaHCO_3_, osmolarity 305–310 mOsm, pH 7.4) at 34 °C for 40 min, and equilibrated at 25 °C for 1 h prior to recordings.

Hippocampal NMDAR-eEPSCs: Whole-cell voltage-clamp recordings (+40 mV) were performed using borosilicate pipettes (4–6 MΩ, P97 Sutter Instruments, USA) filled with intracellular solution (in mmol/L: 115 CsMeSO_4_, 20 CsCl, 10 HEPES, 2.5 MgCl_2_, 4 Na_2_-ATP, 0.4 Na_3_-GTP, 0.6 EGTA, 5 QX-314, and 0.1 Spermin-C14; pH 7.3). ACSF was supplemented with 1 μmol/L TTX, 100 μmol/L PTX, and 10 μmol/L NBQX to isolate NMDAR-mediated currents, as described previously^36^. Schaffer collateral fibers were stimulated (0.1 ms pulses, 1.5 × –2 × threshold) using tungsten electrodes. Signals were acquired at 20 kHz (3 kHz low-pass filter) using an Axon 700B amplifier and a 1440A digitizer (Molecular Devices, USA). The cell membrane potential was clamped at +40 mV during the recording of NMDAR-mediated excitatory postsynaptic currents (NMDAR-eEPSC).

Cultured Neuron Recordings: Whole-cell patch-clamp was conducted using an EPC10 amplifier (HEKA, Germany) with pipettes (3 MΩ) containing either: 1) Action potential recording (in mmol/L): 145 K^+^-gluconate, 3 KCl, 2 MgCl_2_, 1 CaCl_2_, 10 EGTA, 10 HEPES, and 2 Na_2_-ATP, (pH 7.25, 290–300 mOsm); 2) NMDA current solution (in mmol/L): 135 CsF, 0.5 MgCl_2_, 11 EGTA, 10 HEPES (pH 7.4, 290–300 mOsm). Neurons were perfused with: 1) Action potential solution (in mmol/L): 145 NaCl, 3 KCl, 2 MgCl_2_, 1 CaCl_2_, 10 glucose, and 10 HEPES, pH 7.4; 2) NMDA current solution (in mmol/L): 150 NaCl, 4 KCl, 2 CaCl_2_, 5 glucose, and 10 HEPBS; pH 8.0 adjusted with NaOH. For NMDA current recordings in HEK293T cells, the internal solution was identical to the neuronal NMDA current internal solution (solution 2). The cells were perfused with (in mM): 150 NaCl, 2.5 KCl, 1 CaCl_2_, and 10 HEPBS (pH 8.0, adjusted with NaOH). The series resistance was compensated (60%–80%). The resting membrane potential (RMP) was recorded for each neuron in the current clamp mode after stabilization.

### 2.14 Calcium imaging

Cortical neurons (10–14 days *in vitro*) were incubated with 2 μmol/L Fura-2 AM (Invitrogen, USA) in HEPES-buffered saline (in mmol/L: 145 NaCl, 3 KCl, 1 MgCl_2_, 2 CaCl_2_, 10 glucose, 10 HEPES; pH 7.4 adjusted with NaOH) for 20 min at 37 °C, as previously described^33^. Following dye removal, intracellular calcium ([Ca^2+^]_i_) was measured ratiometrically using a MetaFluor imaging system (Molecular Devices). The neurons were transferred to a recording chamber and imaged under dual-wavelength excitation (340/380 nm) at 1 Hz. The 340/380 nm fluorescence ratio was calculated to quantify the [Ca^2+^]_i_ dynamics.

### 2.15 Plasmids and transfection

*Glua1/Glua2* and *Glun1*/*Glun2a* (*Glun2b*) plasmids were cotransfected to HEK293T cell at a 1:1 ratio using Lipofectamine™ 2000 transfection reagent (ThermoFisher, Cat# 11668019, USA). *Glun1*/*Glun2a* (*Glun2b*)/*Psd95* plasmids were cotransfected at a 1:1:1 ratio. Glun2a-C-mCherry-Cry2 (kindly provided by Prof. Chen Zhang, Capital Medical University)^37^ and *Psd95* were cotransfected at a 1:1 ratio.

### 2.16 Western blotting

Briefly, the cortex was collected and homogenized on ice in RIPA buffer (Sigma). The supernatant was collected following centrifugation (10,000×*g*, 10 min). The protein concentration was determined with the BCA protein Assay kit (Generay Biotechnology, Shanghai, China). Protein samples were loaded onto 4%–20% SDS-PAGE gels (Bio-Rad, Cat# 4568094, China) and transferred to a polyvinylidene difluoride membrane (Bio-Rad, Cat# 1620177, China). Primary antibodies, including anti-GluN1 (Proteintech, Cat# 27676-1-AP, USA), anti-GluN2A (Abcam, Cat# ab124913, USA), anti-GluN2B (Abcam, Cat# ab183942, USA), anti-GluA1 (Abcam, Cat# ab183797, USA), anti-GluA2 (Abcam, Cat# ab206293, USA), anti-PSD95 (Abcam, Cat# ab18258, USA), were incubated with the membrane overnight at 4 °C, then following the corresponding second antibodies incubating for 2 h at room temperature. The protein band was detected by chemiluminescence assay and ImageJ software. All proteins were normalized by *β*-actin (Proteintech, 20536-1-AP).

### 2.17 Statistical analysis

All statistical analyses were performed using SPSS 21.0 software (SPSS, Inc., Chicago, IL, USA). All data are expressed as mean ± standard error of mean (SEM). For normally distributed data, differences were evaluated using one-way or two-way analysis of variance (ANOVA), coupled with Tukey's multiple comparison test. Differences in non-normal distributions were evaluated using the Kruskal–Wallis and Dunn–Bonferroni tests. Statistical significance was set at ∗*P* < 0.05; ∗∗*P* < 0.01; ∗∗∗*P* < 0.001.

## 3 Results

### 3.1 YZG-331 ameliorates cognitive deficits in anesthesia/surgery mice

In accordance with a previous study^29^, the protocol of right carotid artery exposure surgery with anesthesia (Fig. 1A) was adopted to replicate the POCD. Cognitive function was assessed using the object recognition test, contextual fear conditioning, and open field test, and conducted according to the timeline shown in Fig. 1A. In the object recognition test model, cognitive and memory impairments are determined by the recognition index and discrimination ratio. The representative trajectories and temporal heatmaps are illustrated in Fig. 1B. Anesthesia/surgery significantly reduced the recognition and discrimination indices, indicating cognitive impairment (Fig. 1B–D). Although YZG-331 alone did not affect cognitive function in the control, administration of YZG-331 at 40 or 80 mg/kg reversed these cognitive deficits in the anesthesia/surgery group (F (5,58) = 9.627, *P* < 0.001; Fig. 1B–D). Contextual fear conditioning test showed YZG-331 (40 and 80 mg/kg) increased movement amplitude and reduced freezing time at both 1 h (F (5,59) = 13.26, *P* < 0.001; Fig. 1E and G) and 24 h post-training (F (5,67) = 6.867, *P* < 0.001; Fig. 1F and H), indicating pronounced learning/memory deficits in anesthesia/surgery mice. YZG-331 (40 and 80 mg/kg) significantly attenuated these impairments in the anesthesia/surgery group (Fig. 1E–H). In contrast, YZG-331 (40 mg/kg) had no effect on the control group (Fig. 1E–H). In the object recognition test and contextual fear conditioning, no intergroup differences in spontaneous locomotor activity were detected in the open field test measures in all of the groups (Supporting information Fig. S1), suggesting that neither anesthesia/surgery nor YZG-331 administration affected motor function or induced anxiety-like behavior. These findings indicate that anesthesia/surgery specifically impairs cognitive and emotional memory functions without affecting locomotor activity or anxiety-like behavior, and that YZG-331 effectively counteracts anesthesia/surgery-induced cognitive deficits.

### 3.2 YZG-331 restored synaptic morphology in anesthesia/surgery mice and attenuates glutamate-induced neuronal morphological alterations

**Figure 2:**
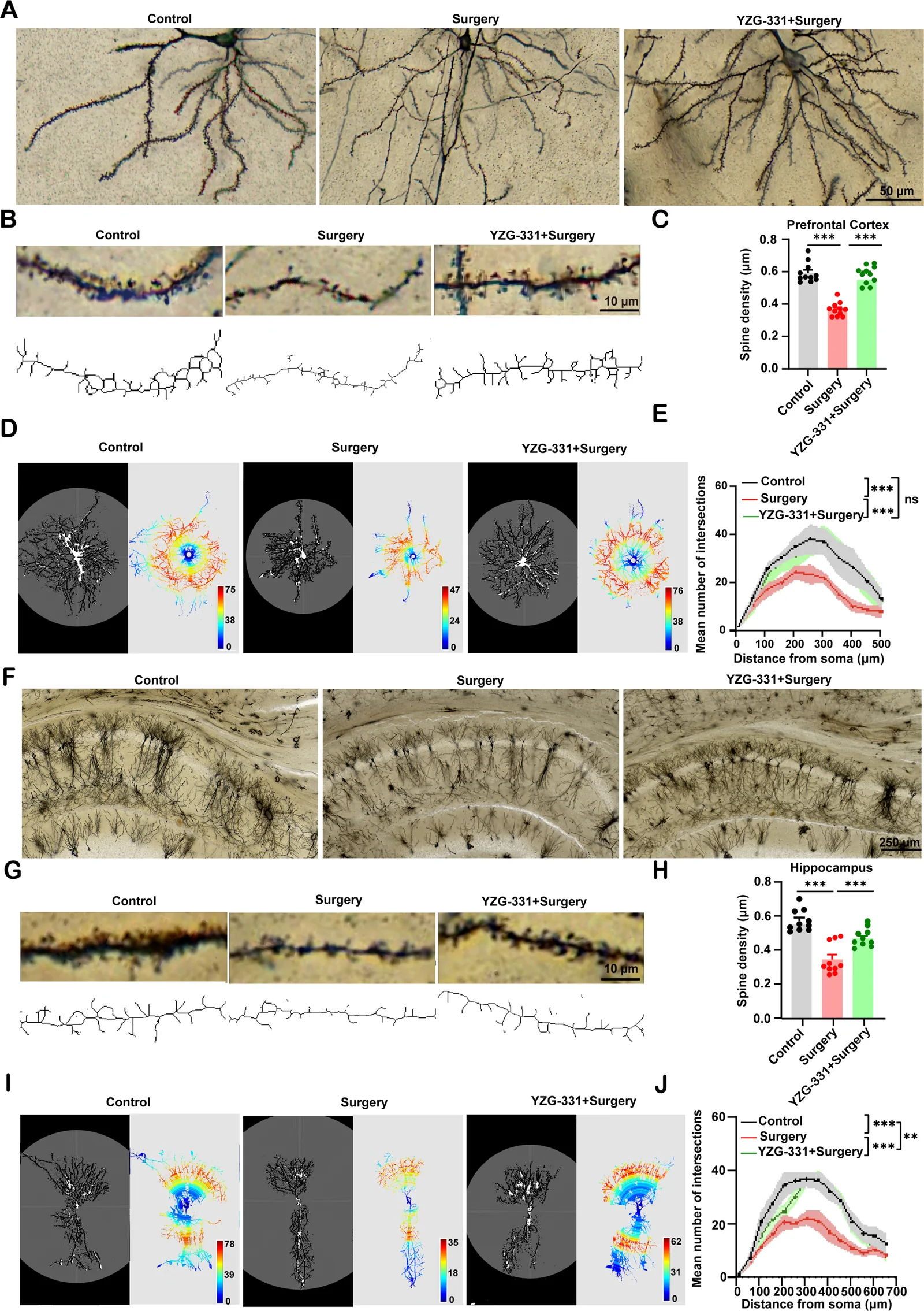
YZG-331 rescued anesthesia/surgery-induced dendritic pathology in the prefrontal cortex and hippocampus. (A, B) Golgi staining of layer III of the mouse prefrontal cortex (A: 200×, *n* = 4, scale bar = 50 μm) and magnification of synapses (B: 1000×, *n* = 4, scale bar = 10 μm). (C) Cortical spine density quantification (control: *n* = 11; anesthesia/surgery: *n* = 10; YZG-331+anesthesia/surgery: *n* = 11). (D, E) Sholl analysis of cortical dendritic complexity in three groups: representative 2D neuronal tracings (left), where branch complexity is indicated by the colors in the trace (right), and the corresponding quantification of dendritic intersections (E; *n* = 6). (F, G) Hippocampal CA1 neurons (F: 200×, scale bar = 250 μm) and spines (G: 1000×, scale bar = 10 μm; *n* = 4). (H) Hippocampal spine density quantification (*n* = 10). (I, J) Hippocampal Sholl analysis in three groups: neuronal tracings (2D, left) accompanied by dendritic intersections graphs (right) (I) and quantification of dendritic intersections (J; *n* = 6). One-way ANOVA followed by Dunnett's test was used for panels C and H, while two-way ANOVA followed by Bonferroni *post hoc* test was used for panels E and J. All data are presented as mean ± SEM. *∗∗P* < 0.01; *∗∗∗P* < 0.001; ns, no significance.

Synaptic plasticity is crucial for cognitive and memory functions. Here we examined the morphology of cortical and hippocampal neurons following anesthesia/surgery. Quantitative analysis revealed anesthesia/surgery-induced reductions in cortical dendritic spine density [F (2,30) = 53.88, *P* < 0.001]; YZG-331 administration significantly restored the spine density (Fig. 2A–C). Sholl analysis further demonstrated a reduction in cortical dendritic branch length and neuronal complexity in anesthesia/surgery mice, as evidenced by reduced dendritic intersections [F (10,176) = 19.97, *P* < 0.001] (Fig. 2D and E). These impairments were reversed by YZG-331 treatment (Fig. 2D and E).

Similarly, hippocampal neurons also exhibited reductions in spine density [F (2,27) = 24.3, *P* < 0.001] and arborization complexity, as well as decreased dendritic intersections [F (13,238) = 47.68, *P* < 0.001] (Fig. 2F–J) in the anesthesia/surgery group. Administration of YZG-331 attenuated these degenerative changes, restoring the hippocampal neuronal morphology to near-control levels. Our findings demonstrate that YZG-331 treatment effectively counteracts anesthesia/surgery-induced structural damage in cortical and hippocampal neurons, establishing its efficacy as a neuroprotective compound against dendritic atrophy and synaptic loss.

### 3.3 YZG-331 attenuates glutamate-induced neuronal morphological and functional alterations by suppressing excitability in cortical neurons

#### 3.3.1 YZG-331 attenuated glutamate-induced morphological alterations

**Figure 3:**
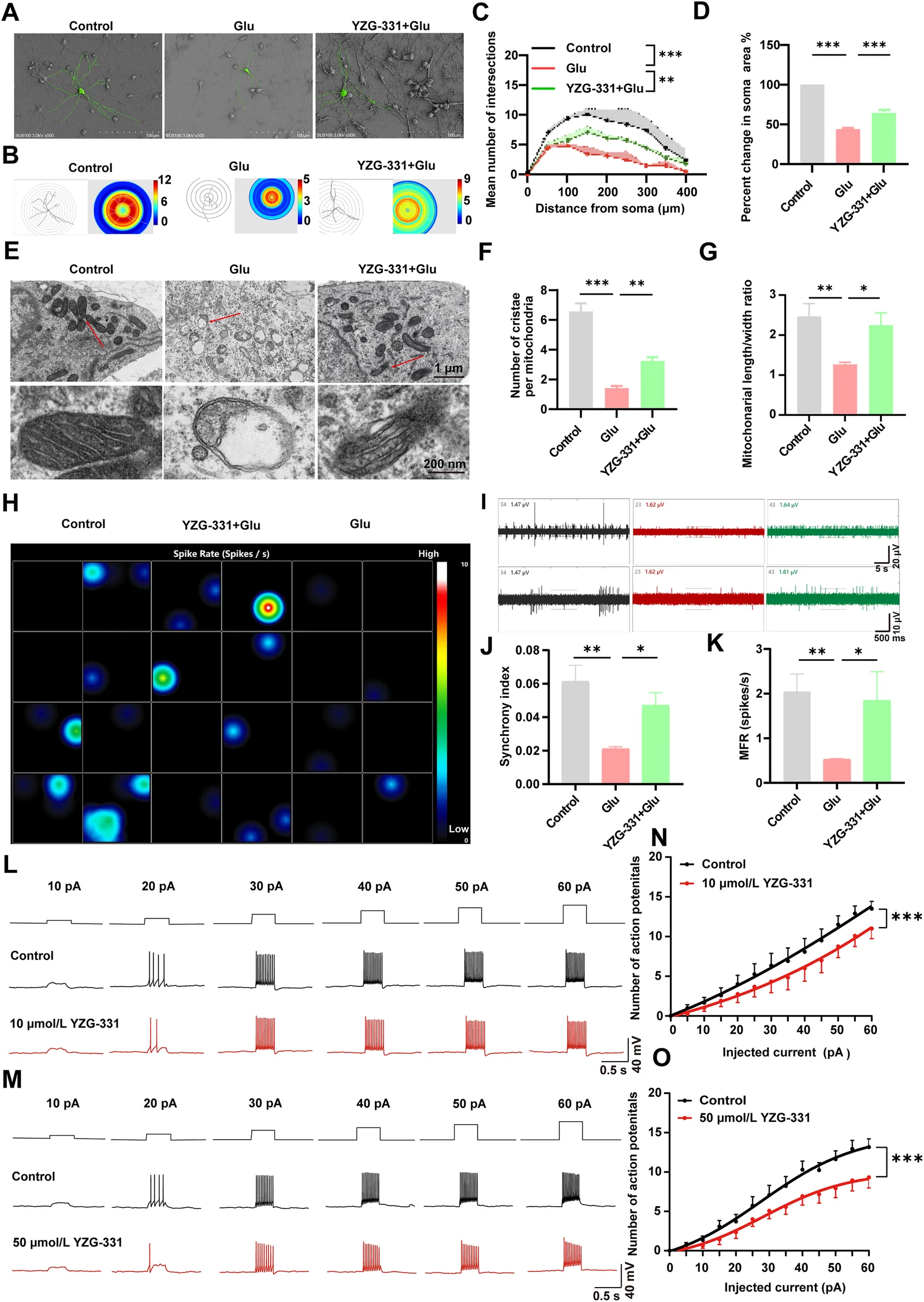
YZG-331 attenuated glutamate-induced excitotoxicity by suppressing the excitability of primary cultured cortical neurons. (A) SEM imaging of cultured cortical neuronal morphology (500×, scale bar = 100 μm). Green cells were labelled as representative neurons. (B, C) Sholl analysis of dendritic complexity of neurons in (A). (B) Concentric circles were drawn with a radius of 5.5 μm steps from the soma (left panel) for the three groups of neurons. The concentric circles are color-coded by the number of intersection points at different distances (right panel, orange = high-density intersection). (C) Quantification of dendritic intersections (*n* = 3). (D) Soma surface area in three groups of neurons (Control/Glu/YZG-331 + Glu: *n* = 21/25/17). (E) Transmission electron microscopy (TEM) ultrastructural analysis of mitochondria in cortical neurons from different groups (left: 5000×, scale bar = 2 μm; right: 20,000×, scale bar = 500 nm). (F) Quantification of mitochondrial cristae (*n* = 3). (G) Mitochondrial morphologic change in three groups of neurons (Control/Glu/YZG-331 + Glu: *n* = 14/12/12). (H) Representative color-coded heatmaps of MEA recordings of cortical neurons from a 24 well MEA plate in different groups. Red and blue indicate high and low firing activities, respectively. (I) Example raw traces of MEA recordings from individual neurons are shown at the top (20 μV, 5 s). The spikes indicate network bursts. The bottom shows a zoom-in image in the red frame (10 μV, 500 ms) (control: black; Glu: red; YZG-331 + Glu: green). (J, K) Quantification of synchrony index (H) and mean firing rate (MFR) of primary cultured cortical neurons (control, *n* = 8; Glu, *n* = 8; YZG-331 + Glu, *n* = 8). (L–O) Current-clamp recordings: typical depolarization-evoked spike trains with 10/50 μmol/L YZG-331 (L, M; 10–60 pA, 10-pA steps) and quantification of action potentials (N, O; *n* = 14) against injected current at above concentration. Two-way ANOVA followed by Bonferroni *post hoc* test was used for C, N and O. One-way ANOVA followed by Dunnett's test was used for D, F, G, J and K. All data are presented as the mean ± SEM. *∗P* < 0.05; *∗∗P* < 0.01; *∗∗∗P* < 0.001.

As previously mentioned, the pathogenesis of POCD involves glutamate toxicity^38^. We then investigated the morphology of cultured cortical neurons following glutamate-induced neuronal excitotoxicity. Consistent with the neuronal morphological changes observed in the POCD animal model, 24 h incubation with glutamate (1 mmol/L) induced significant morphological alterations, characterized by reduced cortical neuronal dendritic complexity and soma shrinkage (SEM quantification, Fig. 3A–D). This insult also induced mitochondrial ultrastructural damage, including swelling, cristae loss, membrane disruption, and vacuole-like alterations (TEM quantification, Fig. 3E–G). Co-treatment with YZG-331 (50 μmol/L) effectively reversed glutamate-induced deficits in dendritic arborization, soma atrophy, and mitochondrial integrity (Fig. 3A–G). These results demonstrate that YZG-331 preserves neuronal architecture and mitochondrial ultrastructure.

#### 3.3.2 YZG-331 mitigates glutamate-induced impairment of cortical neuronal function

Neuronal excitability is one of the key mechanisms underlying POCD^39^. The spontaneous activity of primary cultured cortical neurons was recorded on Microelectrode array (MEA) over 7–10 days *in vitro* (Fig. 3H and J). Developing synaptic connections form functional neuronal networks characterized by propagating spontaneous activity, network bursts, and synchronized firing patterns (Fig. 3H and J). MEA recordings confirmed stable network firing activity in the control cultures for over 72 h. Glutamate exposure (1 mmol/L, 1 h) significantly suppressed the synchronized network firing patterns and reduced the mean firing rate (MFR) (Fig. 3H–K, Supporting information Fig. S2A). YZG-331 treatment partially restored spontaneous network activity, with significant recovery observed after 24 h of incubation (Fig. S2B). Furthermore, YZG-331 directly inhibited the intrinsic neuronal excitability (Fig. 3L–O). This was evidenced by its concentration-dependent inhibition of the frequency of depolarization-induced action potentials (evoked by 10–60 pA current steps; 41% reduction at 50 μmol/L, *P* < 0.001; Fig. 3L–O). These findings establish a dual neuroprotective mechanism for YZG-331, which functions by mitigating glutamate-induced network desynchronization and directly suppressing intrinsic neuronal excitability.

### 3.4 Proteomic profiling reveals YZG-331-mediated neuroprotection *via* NMDA receptor modulation

**Figure 4:**
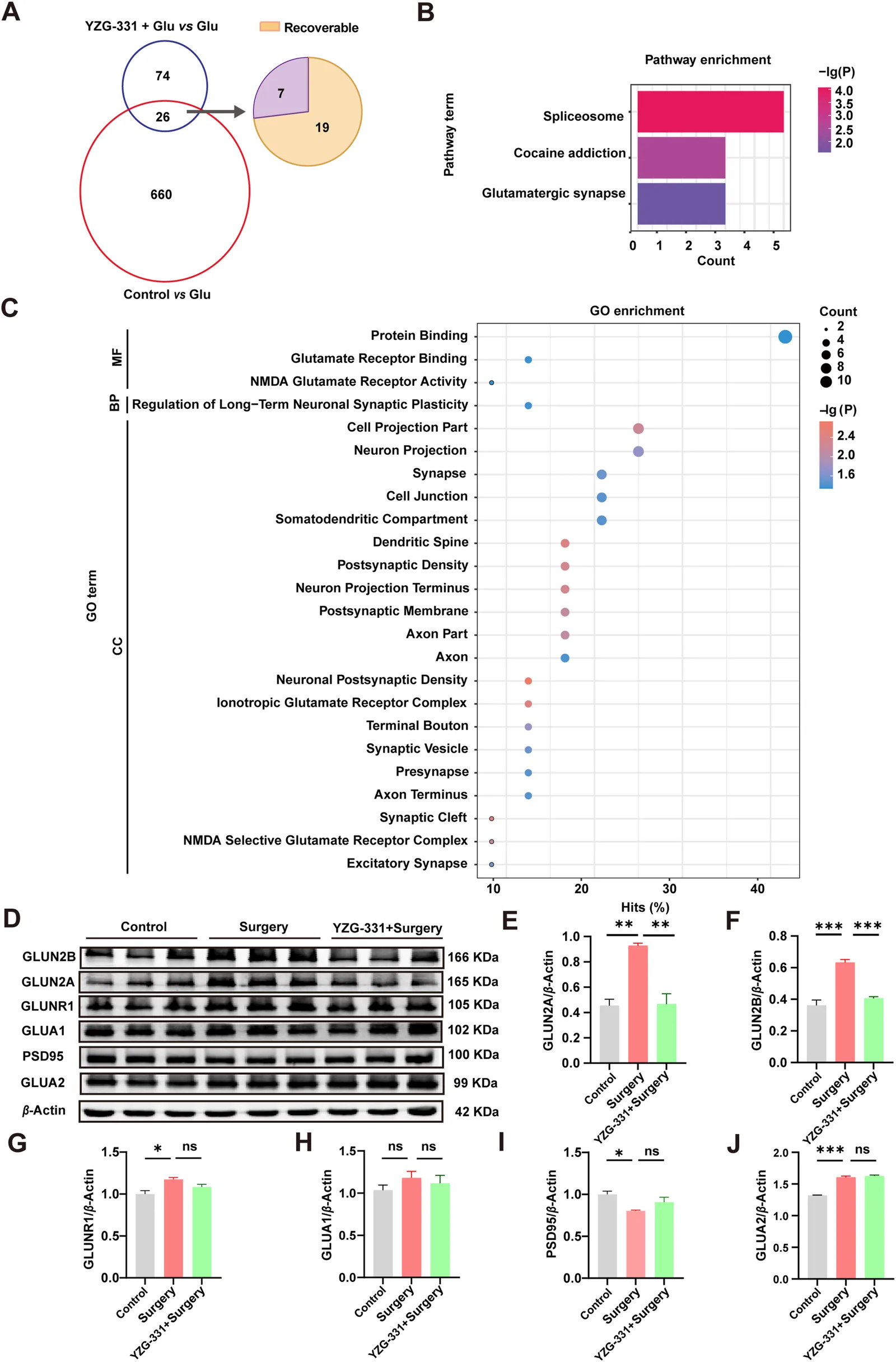
Proteomic Profiling identified NMDAR modulation as the mechanism of YZG-331 neuroprotection. (A) Hierarchical clustering of 26 co-DEPs in the experimental groups. (B) KEGG pathway enrichment analysis of glutamate-induced DEPs highlighted their significant involvement in glutamate-induced cell toxicity. (C) GO term enrichment demonstrates tripartite regulation of NMDAR complexes (molecular function), postsynaptic membranes (cellular component), and neuroplasticity (biological process). (D) Representative images of Western blots for the expression of GLUN2A, GLUN2B, GLUNR1, GLUA1, PSD95, and GLUA2 in the cortex of different Groups. (E–J) Quantitative analysis of the results. (*n* = 3 mice for each group: control, anesthetic/surgery, and YZG-331) One-way ANOVA followed by Dunnett's test was used for E–J. All data are presented as the mean ± SEM. *∗P* < 0.05; *∗∗P* < 0.01; *∗∗∗P* < 0.001; ns, no significance.

Proteomic analysis of primary cortical neurons under three conditions (control, glutamate, and YZG-331 + glutamate) identified 5059 proteins. Partial Least Squares Discriminant Analysis (PLS-DA) revealed distinct intergroup clustering (Supporting information Fig. S3A), as previously described^40^. Volcano plots identified differentially expressed proteins (DEPs; logFC ≥1.2 or ≤ 0.8; *∗P* < 0.05; Fig. S3B), yielding 660 DEPs between control and glutamate groups, 74 DEPs between glutamate and YZG-331 with glutamate and 26 co-regulated DEPs (co-DEPs). Among these co-DEPs, 19 proteins in the YZG-331 group exhibited expression profiles resembling those of the controls (Fig. 4A). Convergent KEGG/GO analyses revealed enriched glutamatergic synapse pathways (Fig. 4B), particularly NMDAR complexes, as key mediators of YZG-331's protection against glutamate-induced toxicity (Fig. 4C). As illustrated in Fig. 4D–J, Western blot analysis demonstrated a significant upregulation of GLUN1, GLUN2A, GLUN2B, and GLUA2 expression in the cortex of the anesthetic/surgery mouse model. Conversely, the expression of PSD95 was notably downregulated in the same model. YZG-331 significantly reversed the upregulation of GLUN2A and GLUN2B induced by anesthetic/surgery without affecting the expression of other proteins. These findings identified NMDAR modulation as the central mechanism underlying the neuroprotective effects of YZG-331 against glutamate toxicity.

### 3.5 YZG-331 attenuates glutamate-induced calcium dysregulation *via* NMDAR modulation

#### 3.5.1 Calcium imaging reveals NMDAR subtype of iGluR inhibition by YZG-331

**Figure 5:**
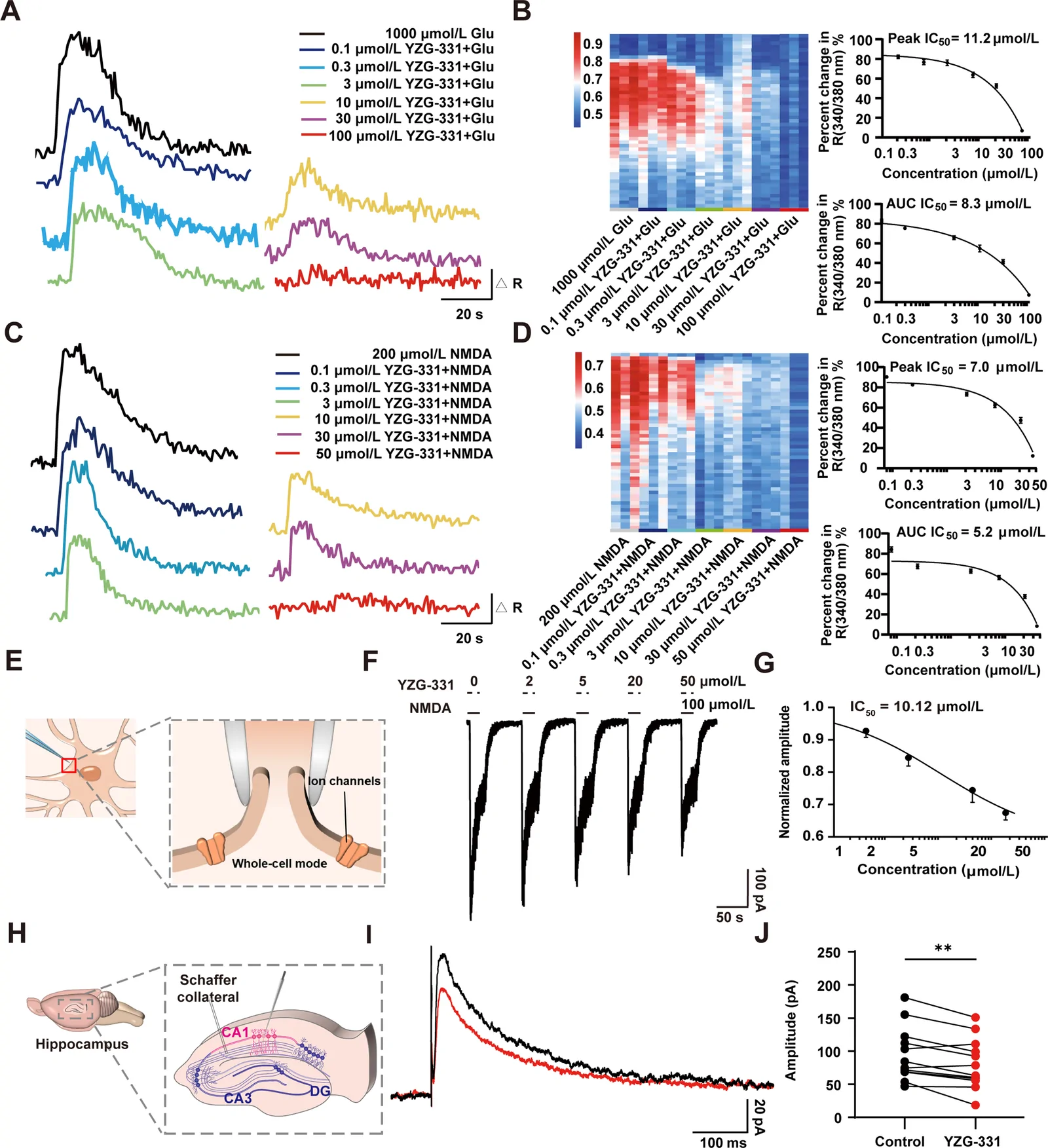
YZG-331 modulates [Ca^2+^]_i_ influx through targeting NMDA receptor targeting. (A) Calcium imaging paradigm for quantifying agonist Glu-induced [Ca^2+^]_i_ upregulation. (B) Concentration-dependent inhibition profiles: heatmaps (left) and normalized dose–response curves (right) of YZG-331 on [Ca^2+^]_i_ peak and AUC for Glu. (C) Calcium imaging paradigm for quantifying agonist NMDA-induced [Ca^2+^]_i_ upregulation. (D) Concentration-dependent inhibition profiles: heatmaps (left) and normalized dose–response curves (right) of YZG-331 on [Ca^2+^]_i_ peak and AUC for NMDA. (E) Schematic diagram of the neuronal whole-cell patch clamp. (F, G) NMDA-evoked current inhibition: representative traces (F) and quantitation (G) showing dose-responsive blockade (IC_50_ = 10.12 μmol/L, *n* = 34 cells). (H–J) Synaptic NMDAR-EPSC modulation: experimental schematic (H), superimposed eEPSC traces at +40 mV (I, control: black; 50 μmol/L YZG-331: red), and amplitude quantification (J, Student's paired two-tailed *t* test, *n* = 12 cells from five mice; *∗∗P* < 0.01). All data are presented as mean ± SEM.

To confirm the proteomic results, we performed functional validation using calcium imaging. YZG-331 suppressed glutamate-induced [Ca^2+^]_i_ overload in a dose-dependent manner (peak IC_50_ = 11.2 μmol/L, area under the curve: AUC IC_50_ = 8.3 μmol/L, Fig. 5A and B), achieving 93% peak and 92% AUC inhibition at 100 μmol/L. The broad-spectrum efficacy of YZG-331 was also demonstrated by its ability to inhibit NMDA-induced [Ca^2+^]_i_ overload (88% peak inhibition, 91% AUC inhibition, IC_50_ = 7.0/5.2 μmol/L, Fig. 5C and D). This inhibition profile reveals that YZG-331 is a novel stabilizer of neuronal calcium homeostasis.

To further substantiate the inhibitory effect of YZG-331 on NMDAR, whole-cell patch-clamp experiments were conducted on cortical neurons to directly verify its antagonistic effect on NMDA receptors. YZG-331 dose-dependently inhibited the NMDAR current, with a 67% reduction in current at 50 μmol/L (Fig. 5E–G). Additionally, hippocampal slice recordings demonstrated 16.5% suppression of YZG-331 on the amplitude of NMDAR-mediated excitatory postsynaptic currents (NMDAR-eEPSCs) (83.5% ± 4.7% of control, Fig. 5H–J), confirming the efficacy of YZG-331 in inhibiting NMDARs at the synaptic level. Taken together, these findings confirm that the NMDAR is the primary mechanism underlying YZG-331's calcium homeostasis.

#### 3.5.2 YZG-331 suppresses AMPA/kainate receptor-mediated calcium influx through NMDARs

**Figure 6:**
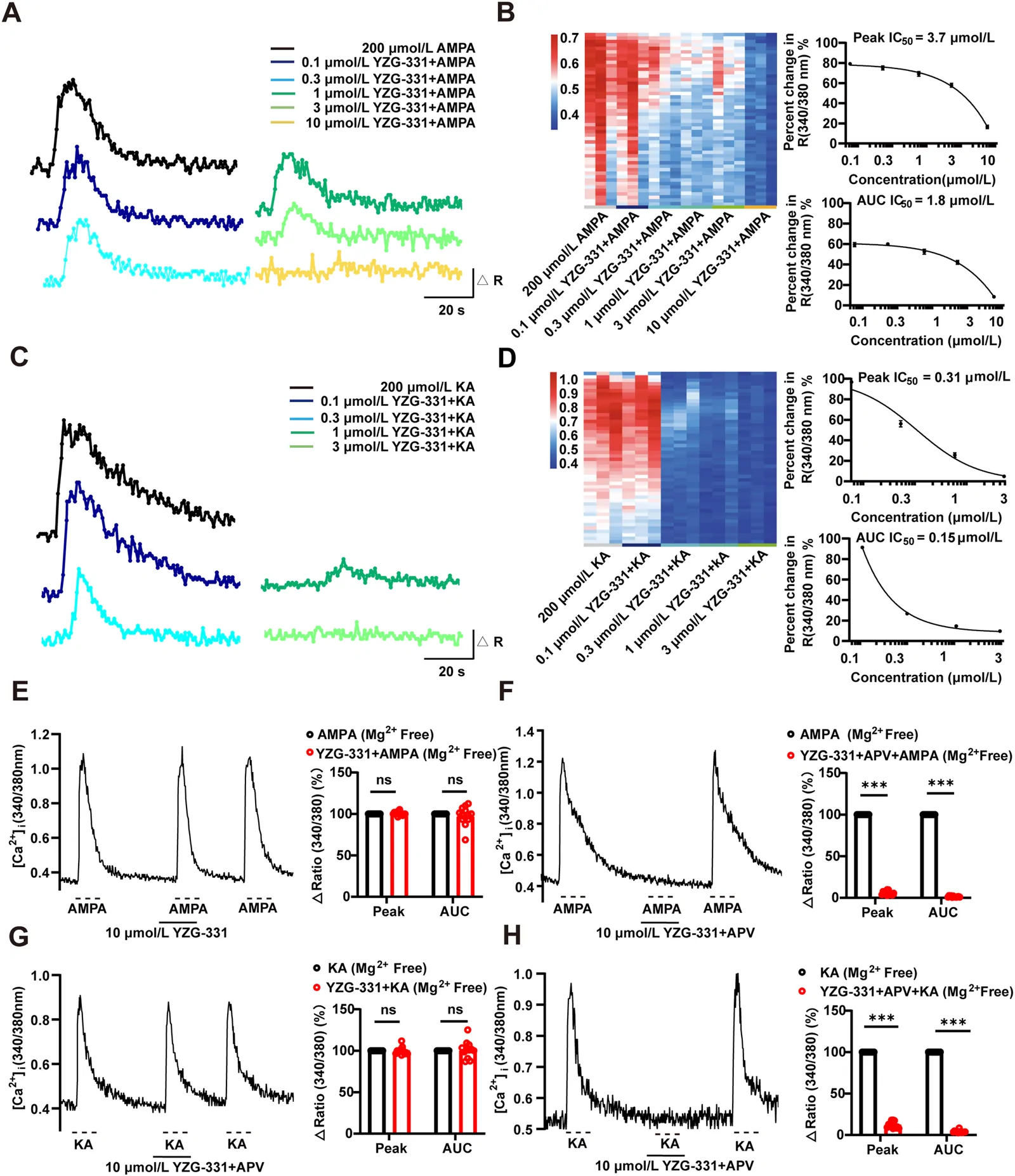
The effect of YZG-331 on AMPA receptor- and kainate receptor-mediated [Ca^2+^]_i_ influx. (A) Calcium imaging paradigm for quantifying agonist AMPA-induced [Ca^2+^]_i_ upregulation. (B) Concentration-dependent inhibition profiles: heatmaps (left) and normalized dose–response curves (right) of YZG-331 on [Ca^2+^]_i_ peak and AUC for AMPA. (C) Calcium imaging paradigm for quantifying agonist KA-induced [Ca^2+^]_i_ upregulation. (D) Concentration-dependent inhibition profiles: heatmaps (left) and normalized dose–response curves (right) of YZG-331 on [Ca^2+^]_i_ peak and AUC for KA. (E, F) Representative traces and quantitative analysis illustrate the effect of YZG-331 on AMPA receptors-mediated elevations in [Ca^2+^]_i_ in neurons treated without or with APV under Mg^2+^-free conditions. (G, H) Representative traces and quantitative analysis illustrate the effect of YZG-331 on kainate receptors-mediated elevations in [Ca^2+^]_i_ in neurons treated without or with APV under Mg^2+^-free conditions. Statistical annotation: data were analyzed using paired *t* test (E–H). All data are presented as the mean ± SEM; ∗∗∗*P* < 0.001.

We next tested whether YZG-331 exerted inhibitory effects on AMPA/kainic acid (KA)-mediated calcium influx. The results displayed that YZG-331 suppressed AMPA-induced [Ca^2+^]_i_ overload in a dose-dependent manner (84% peak inhibition, 92% AUC inhibition, IC_50_ = 3.7/1.8 μmol/L, Fig. 6A and B), as well as KA-induced [Ca^2+^]_i_ overload (95% peak inhibition, 99% AUC inhibition, IC_50_ = 0.31/0.15 μmol/L, Fig. 6C and D). However, under Mg^2+^-free conditions, YZG-331 lost efficacy against AMPA/KA-induced Ca^2+^ influx (Fig. 6E and G). Meanwhile, application of the NMDAR antagonist (APV) abolished AMPA/KA-induced calcium influx in the presence of YZG-331 (Fig. 6F and H). These results indicate that AMPA/KA-induced calcium influx relies on NMDARs. We then examined whether YZG-331 acts directly on AMPARs through the heterologous expression of AMPA receptor subunits (*Glua1* and *Glua2*) in the HEK293T cells. YZG-331 does not alter glutamate or AMPA-evoked [Ca^2+^]_i_ (in the presence of 1 mmol/L Mg^2+^, Supporting information Fig. S4A and S4B). Additionally, YZG-331 did not change the desensitization kinetics (*τ* = 4.3 ± 0.2 ms *vs.* control 4.1 ± 0.3 ms) and the amplitude of GLUA2 currents (98.7 ± 3.1% of baseline) (Fig. S4C and S4D). Collectively, these findings suggest that the suppression of YZG-331 on AMPA/KA-induced calcium influx relies on NMDARs.

### 3.6 YZG-331 potentiates Mg^2+^ blockade of NMDAR

**Figure 7:**
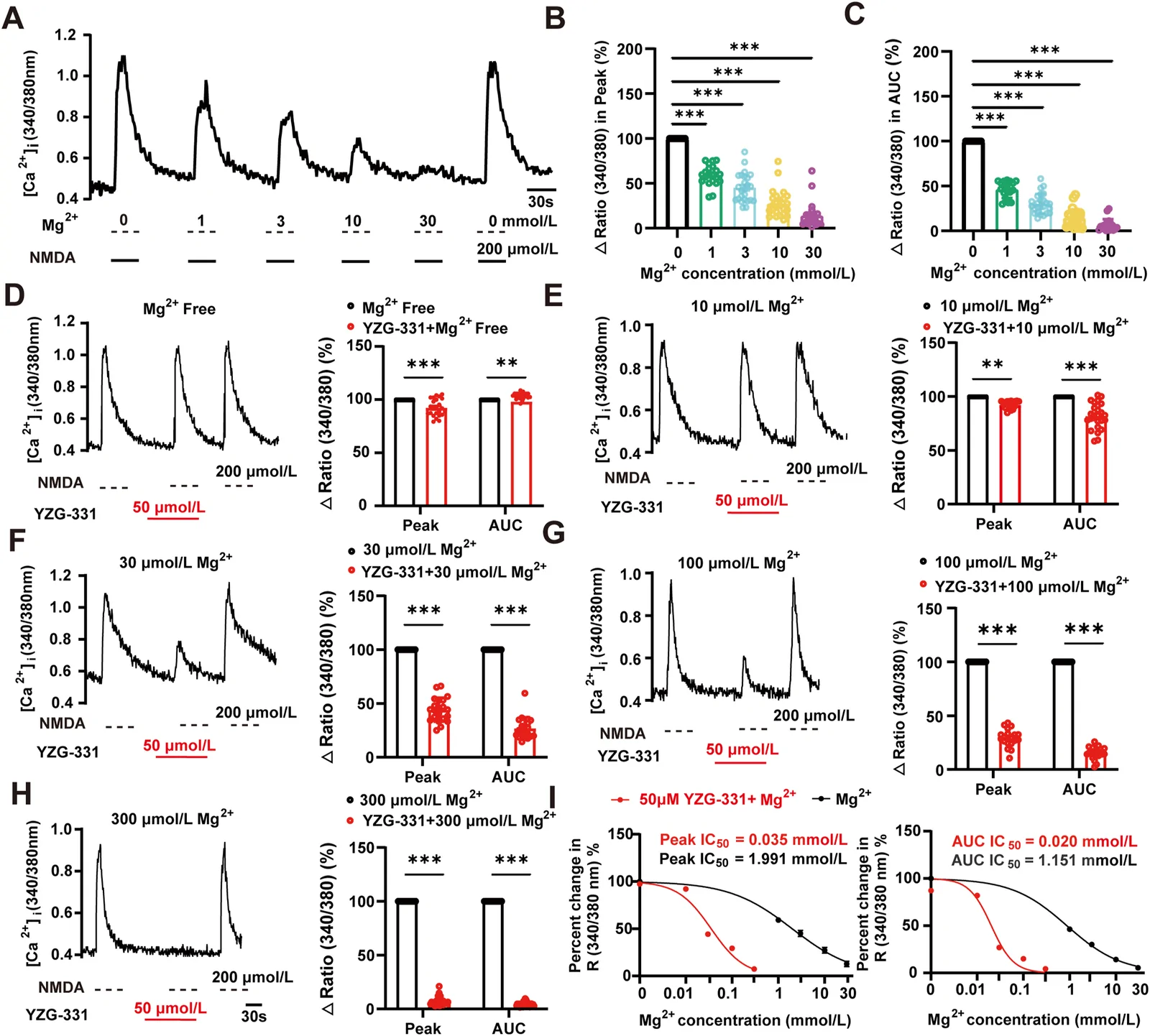
YZG-331 potentiates Mg^2+^ blockade of NMDAR. (A) Mg^2+^-dependent suppression of NMDA-evoked [Ca^2+^]_i_ upregulation in cortical neurons (200 μmol/L NMDA). (B, C) Quantitative inhibition of peak amplitude (peak, B) and integrated calcium load (AUC, C) by Mg^2+^ (0–30 mmol/L). (D–H) Mg^2+^-concentration gradient (0–300 μmol/L) revealing YZG-331's (50 μmol/L) conditional efficacy: inhibition achieved at 100 μmol/L Mg^2+^ in the presence of YZG-331 (peak 29.3 ± 2.1%, AUC 18.7 ± 1.8%). (I) Leftward shift of Mg^2+^ dose–response curves with YZG-331 (50 μmol/L) co-application (peak IC_50_ = 0.035 mmol/L *vs.* control 1.99 mmol/L; AUC 0.02 mmol/L *vs.* 1.15 mmol/L, *n* = 25). Statistical annotation: data were analyzed using one-way ANOVA followed by Dunnett's test (B, C), paired *t* test (D–H), and two-way ANOVA followed by Bonferroni *post hoc* test (I). All data are presented as the mean ± SEM; *∗∗P* < 0.01; *∗∗∗P* < 0.001.

Given that YZG-331 lost its inhibitory effect on AMPA/KA induced calcium influx without magnesium, we assume that magnesium is involved in the inhibition of calcium influx by YZG-331. To verify this hypothesis, Mg^2+^ was applied to test its inhibition of NMDA-mediated calcium influx. Mg^2+^ alone induced concentration-dependent suppression on NMDA-evoked [Ca^2+^]_i_ upregulation (peak: 59.1 ± 2.5% to 12.8 ± 2.9%; AUC: 46.2 ± 2.2% to 5.5 ± 1.3% at 1–30 mmol/L Mg^2+^, Fig. 7A–C). Strikingly, Mg^2+^-free conditions abrogated YZG-331's inhibitory efficacy on NMDA responses (Fig. 7D ), while increasing extracellular Mg^2+^ (0.01–0.3 mmol/L) progressively enhanced YZG-331 (50 μmol/L)-mediated suppression (Fig. 7E–H). YZG-331 significantly enhanced the inhibitory potency of Mg^2+^ on NMDAR, with a 28.6-fold increase in peak inhibition (IC_50_ = 0.035 mmol/L compared to control 1.99 mmol/L) and a 50-fold increase in AUC inhibition (IC_50_ = 0.02 mmol/L compared to control 1.15 mmol/L). This indicates the allosteric potentiation effect of YZG-331 on the magnesium blockade of NMDAR (Fig. 7I). This synergistic mechanism of magnesium with YZG-331 inhibits pathological calcium overload, establishing YZG-331 as a novel pharmacodynamic inhibitor of the NMDAR.

**Figure 8:**
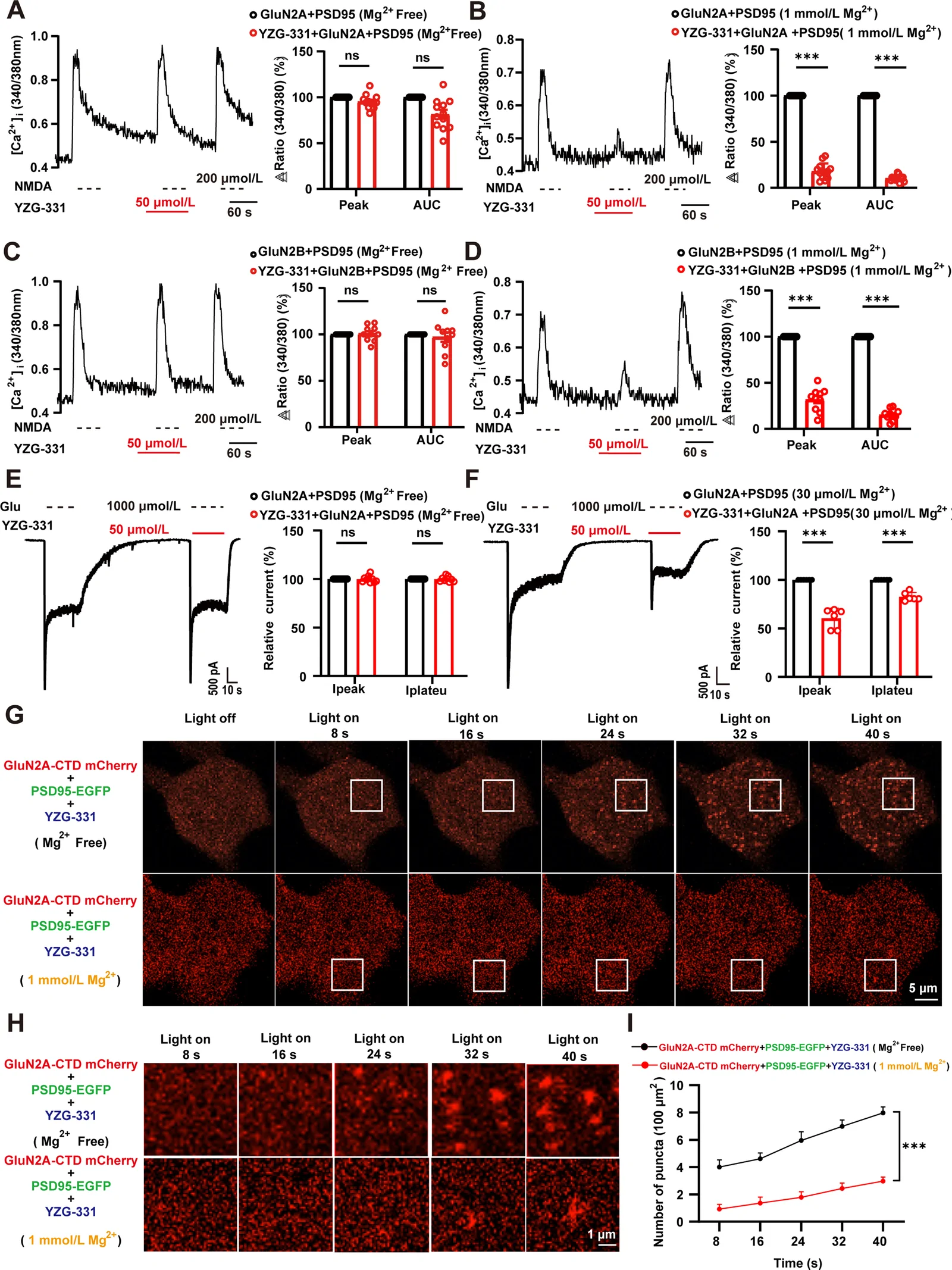
Mg^2+^-coordinated allosteric inhibition of NMDAR by YZG-331. YZG-331 effects on NMDA-induced [Ca^2+^]_i_ responses in HEK293 cells expressing recombinant NMDAR. (A) GLUN1/GLUN2A/PSD95 without Mg^2+^ (*n* = 11). (B) GLUN1/GLUN2A/PSD95 with 1 mmol/L Mg^2+^ (*n* = 12). (C) GLUN1/GLUN2B/PSD95 without Mg^2+^ (*n* = 10). (D) GLUN1/GLUN2B/PSD95 with 1 mmol/L Mg^2+^ (*n* = 10). (E, F) Whole-cell patch-clamp recordings of NMDA receptor currents. (E) GLUN1/GLUN2A/PSD95 without Mg^2+^ (*n* = 6). (F) GLUN1/GLUN2A/PSD95 with 30 μmol/L Mg^2+^ (*n* = 6). (G–I) Optogenetic reconstitution of GLUN2A-CTD/PSD95 phase separation. (G) Time-lapse imaging of GLUN2A-CTD-mCherry condensates under blue light stimulation (8–40 s; top: no Mg^2+^, bottom: with 1 mmol/L Mg^2+^). (H) Magnified view of the boxed regions in (G). (I) Quantification of condensates (>0.5 μm²) showing Mg^2+^-dependent suppression by YZG-331 (black: control, *n* = 14; red: YZG-331 + Mg^2+^, *n* = 12; ∗∗∗*P* < 0.001, two-tailed *t* test). Data were analyzed using a paired *t* test for A–F and two-way ANOVA followed by Bonferroni *post hoc* test for I. All data are presented as the mean ± SEM; *∗∗∗P* < 0.001; ns, no significance.

To elucidate the molecular mechanism underlying YZG-331-mediated NMDAR regulation, we conducted functional studies using HEK293T cells overexpressing recombinant NMDAR subunits. Given that PSD95 scaffolds NMDAR at synaptic sites *via* PDZ domain interactions^34^, we co-expressed *Psd95* with *Glun1*/*Glun2A* in HEK293T cells. Strikingly, YZG-331 exhibited no inhibitory effect in the Mg^2+^-free medium (Fig. 8A), while it completely abolished NMDA-evoked [Ca^2+^]_i_ increases in the presence of 1 mmol/L Mg^2+^ (Fig. 8B). This Mg^2+^-dependent inhibition pattern was also observed in GLUN1/GLUN2B/PSD95-expressing cells (Fig. 8C and D). Electrophysiological recordings further confirmed that YZG-331 significantly attenuated NMDAR-mediated currents, specifically when Mg^2+^ was present (Fig. 8E and F). These data establish that the NMDAR antagonism exerted by YZG-331 requires the presence of Mg^2+^.

To further characterize this molecular interplay, we employed optogenetic clustering assays with mCherry-Cry2-tagged to the C-terminal domain (CTD) of *Glun2A*. In the presence of YZG-331, large CTD-PSD95 aggregates were observed to form in a time-dependent pattern in the absence of Mg^2+^ (Fig. 8G and H). Remarkably, Mg^2+^ (1 mmol/L) substantially reduced the number of macromolecular assemblies (Fig. 8G and H), with quantitative analysis confirming a significant decrease in droplet numbers compared to Mg^2+^-free conditions (*P* < 0.001; Fig. 8I). Collectively, these findings indicate that YZG-331 exerts regulatory effects on NMDAR by enhancing Mg^2+^-dependent channel blockade. This synergy between the pharmacological potentiation of Mg^2+^ blockade on NMDAR assembly provides a novel therapeutic paradigm for mitigating NMDAR-mediated excitotoxicity.

## 4 Discussion

POCD presents a significant clinical challenge as there are currently no effective preventive or therapeutic agents available for this condition. Our findings demonstrate that the adenosine analogue YZG-331 effectively mitigates cognitive deficits and dendritic spine loss in a POCD mouse model. Building upon its known sedative properties, we elucidate a novel neuroprotective mechanism whereby YZG-331 alleviates POCD-induced neuronal impairments by specifically modulating NMDAR function. Crucially, we demonstrate that YZG-331 suppresses NMDAR by enhancing the potency of Mg^2+^. The targeted inhibition of NMDAR provides a compelling molecular basis for the restoration of cognitive function and synaptic integrity. This establishes YZG-331 as a promising therapeutic candidate for POCD and highlights the critical interplay between Mg^2+^ and NMDAR dynamics for future modulator development. Our findings advance the understanding of therapeutic interventions for POCD and provide a novel approach to modulating NMDAR signaling.

### 4.1 Synaptic reconfiguration as a therapeutic target

Clinical studies have identified POCD as a critical clinical challenge during the perioperative period. Cognitive impairment represents the most prominent symptom of POCD, often persisting for extended durations. Synaptic plasticity is a critical determinant of cognitive function, and its disruption is a hallmark of POCD. The observed dendritic atrophy and impaired Sholl intersections (Fig. 2) in the POCD models confirm the previous evidence that cognitive decline originates from excitatory synapse destabilization^21^^,^^41^. Here, we demonstrate that YZG-331, a blood–brain barrier-penetrant adenosine analog, significantly rescues anesthesia/surgery-associated cognitive deficits and reverses synaptic structural deficits in the cortex and hippocampus of anesthesia/surgery mice. Notably, YZG-331 demonstrates an exceptional safety profile and no dependence liability, attributes that are critical for perioperative pharmacological interventions.

### 4.2 Mechanistic insights into Mg^2+^ modulation

At the molecular level, our investigations revealed YZG-331's neuroprotective efficacy against glutamate-induced excitotoxicity, a pathological hallmark of POCD^30^. YZG-331 significantly preserved dendritic architecture and mitochondrial integrity (Fig. 3), whereas proteomic profiling identified NMDAR modulation as a key mechanistic component (Fig. 4). The centrality of the NMDAR to excitotoxic pathology^42^^,^^43^ has been reported previously. This pathway may represent a maladaptive response to neuronal stress, potentially modulating NMDAR surface expression or assembly to alter excitatory signaling. YZG-331 exhibited dose-dependent inhibition of NMDA-mediated calcium influx while displaying NMDAR subtype expression specificity, with AMPA/kainate receptor activity remaining unaffected. These functional findings were consistent with the protein expression data, thereby confirming the role of YZG-331 in modulating NMDAR signaling. Collectively, these findings indicate that the neuroprotective effects of YZG-331 are mediated through functional normalization of NMDAR activity, which in turn halts dendritic spine loss and promotes synaptic recovery. Furthermore, YZG-331 reversed the upregulation of GLUN2A/2B induced by POCD, reflecting restoration of synaptic homeostasis—a phenomenon that is fully consistent with its inhibitory action on NMDAR function. However, further exploration is required to elucidate the mechanisms by which YZG-331 modulates the protein expression of NMDAR subunits.

NMDAR is intrinsically regulated by Mg^2+^ through high-affinity binding within its channel pore, establishing a critical physiological safeguard against calcium overload^44^^,^^45^. Patients with GRIN mutations exhibiting Mg^2+^ blockade dysfunction experience neuronal developmental delay, intellectual disability, and epilepsy^46^, ^47^, ^48^. Therefore, Mg^2+^ is often used for neuronal disorders, including migraine, depression, epilepsy, stroke, and Alzheimer's disease^49^^,^^50^. Developing drugs that enhance the efficacy of Mg^2+^ is an effective approach for developing NMDAR drugs. Our data reveal that YZG-331 acts through this groundbreaking mechanism by enhancing the voltage-dependent Mg^2+^ blockade efficacy by 50-fold (in AUC Fig. 7). This is an unprecedented pharmacological property among NMDAR antagonists. Electrophysiological evidence of reduced NMDAR currents and synaptic transmission (Fig. 8E and F, Fig. 5E–J) supports this novel modulatory effect. This distinguishes YZG-331 from conventional NMDAR antagonists (*e.g.*, MK-801), which compete with the Mg^2+^ binding site^45^^,^^51^^,^^52^, suggesting a novel mechanism of NMDAR modulation by YZG-331 rather than direct channel antagonism. This Mg^2+^-sensitizing property holds particular therapeutic promise, given the narrow physiological range of serum Mg^2+^ (0.75–1.0 mmol/L) and toxicity risks at supra-physiological concentrations^53^^,^^54^. By enhancing endogenous Mg^2+^ efficacy rather than competing with it, YZG-331 circumvents the limitations of current NMDAR-targeted therapies while maintaining homeostatic ion balance. The clinical implications extend beyond POCD to various neuro-pathologies characterized by GRIN mutations^46^.

PSD-95, the principal organizer of postsynaptic nanodomains, orchestrates NMDAR synaptic localization through high-affinity PDZ domain interactions^55^. PSD-95 promotes the clustering of NMDAR at synapses^56^ and reduces their desensitization^57^. Additionally, PSD-95 was reported to form a liquid condensate with NMDAR and other postsynaptic proteins^21^^,^^58^^,^^59^. YZG-331 disrupts this synaptic nano-architecture through interaction with NMDAR in the presence of Mg^2+^, thereby reducing NMDAR membrane density (Fig. 8). This phenomenon is highly intriguing. The disaggregation of NMDARs may suggest additional regulatory mechanisms for YZG-331 beyond its enhanced affinity for Mg^2+^. However, the mechanisms underlying this phenomenon require further investigation.

### 4.3 Perspectives and limitations

While our findings establish YZG-331 as a first-in-class Mg^2+^ efficacy enhancer, several mechanistic questions warrant further investigation. Behavioral and functional tests should be performed at different time points to evaluate the effects of YZG-331 on postoperative agitation and delirium. The precise structural interactions between YZG-331, Mg^2+^, and NMDAR subunits require elucidation using Cryo-EM studies and molecular dynamics simulations. Additionally, potential post-translational modifications that influence receptor-scaffold interactions should be explored using phosphoproteomic and palmitoylomic analyses. Moreover, translational studies using GRIN mutation models could validate the therapeutic potential of YZG-331 in neurological disorders.

## 5 Conclusions

This study identified YZG-331 as a novel pharmacological agent that synergizes with endogenous Mg^2+^ to mitigate NMDAR hyperactivation, thereby reducing synaptic impairment. Its ability to enhance magnesium blockade and restore synaptic architecture positions YZG-331 as a groundbreaking therapeutic candidate for POCD and related neurocognitive disorders. Insights into the modulation of NMDAR–Mg^2+^–YZG-331 interaction will open new avenues for targeting NMDAR and magnesium interactions in pathologies associated with excitotoxicity.

## Author contributions

Wei Zhang was the principal architect of the project, devising the experiments and revising the manuscript. Han Guo conducted the behavioral experiments, calcium imaging experiments, scanning electron microscopy, transmission electron microscopy, Golgi staining, and microelectrode array experiments, as well as drafting the manuscript. Jiaojiao Zhao performed the patch-clamp experiments on cell membranes. Qinghui Guo carried out the phase separation experiments. Siruan Chen, Keying Tian, Yuhan Ge, Wenyan Guo, Xingchen Lin, Hui Bai, Lei Yang, Zuxiao Yang, Xia Qin, Panpan Zhang, Ruifang Zheng and Zhanfeng Jia were pivotal researchers who performed data analysis. Tengfei Ji, Zuxiao Yang, Dezhi Kong, and Yun Stone Shi provided guidance on experimental design and revising manuscript. All authors have read, edited, and approved the final manuscript.

## Conflicts of interest

The authors declare no conflict of interest.

## Appendix A Supporting information

The following is the Supporting information to this article.
