# Supplementary material for: Neuroprotection by YZG-331 in postoperative cognitive dysfunction via Mg2+-dependent blockade of NMDA receptors
Source: Acta Pharm Sin B. 2026 Jul 20;16(9):5995–6012. doi: 10.1016/j.apsb.2026.07.025 (PMC13590013; doi:10.1016/j.apsb.2026.07.025)
Supplement: Supplementary file 1 [file mmc1.pdf]

Supporting Information for

Original article

Neuroprotection by YZG-331 in postoperative cognitive dysfunction *via* Mg^{2+}-dependent blockade of NMDA receptors

Han Guo[a,b], Jiaojiao Zhao[a], Keying Tian[a], Qinghui Guo[a], Siruan Chen[a], Yuhan Ge[c,d], Wenyan Guo[a], Xingchen Lin[d,e], Hui Bai[f], Lei Yang[g], Lei Yang[a], Xia Qin[a], Panpan Zhang[a], Zhanfeng Jia[h], Ruifang Zheng[i], Zuxiao Yang[a,*], Yun Shi[c,d,*], Dezhi Kong[a,*], Tengfei Ji[j,i,*], Wei Zhang[a,*]

[a]*Department of Pharmacology of Chinese Materia Medica, Institution of Chinese Integrative Medicine, The Key Laboratory of Tranquilizing TCM, Hebei Provincial Administration of Traditional Chinese Medicine, The Key Laboratory of Neural and Vascular Biology, Ministry of Education, Research Unit of Digestive Tract Microecosystem Pharmacology and Toxicology, Chinese Academy of Medical Sciences, Hebei Medical University, Shijiazhuang 050017, China*

[b]*Department of Oral Medicine, The Second Hospital of Hebei Medical University, Shijiazhuang 050000, China*

[c]*Model Animal Research Center, Medical School, Nanjing University, Nanjing 210032, China*

[d]*Guangdong Institute of Intelligence Science and Technology, Zhuhai 519031, China*

[e]*Center for Biological Science and Technology, Advanced Institute of Natural Sciences, Beijing Normal University, Zhuhai 519087, China*

[f]*Department of Cardiac Ultrasound, The Second Hospital of Hebei Medical University, Shijiazhuang 050000, China*

[g]*School of Basic Medical Sciences, Capital Medical University, Beijing 100069, China*

[h]*Department of Pharmacology, Hebei Medical University, Shijiazhuang 050017, China*

[i]*Xinjiang Key Laboratory of Uygur Medical Research, Xinjiang Institute of Materia Medica, Urumqi 841100, China*

[j]*Institute of Materia Medica, Chinese Academy of Medical Sciences and Peking Union Medical College, Beijing 100050, China*

Received 2 September 2025; received in revised form 18 January 2025; accepted 17 February 2026

[*]Corresponding authors.

E-mail addresses: weizhang@hebmu.edu.cn (Wei Zhang), jitf@imm.ac.cn (Tengfei Ji), konfdezhi@hebmu.edu.cn (Dezhi Kong), shiyun@gdiist.cn (Yun Shi), yangzuxiao@hebmu.edu.cn (Zuxiao Yang).

Supporting figures

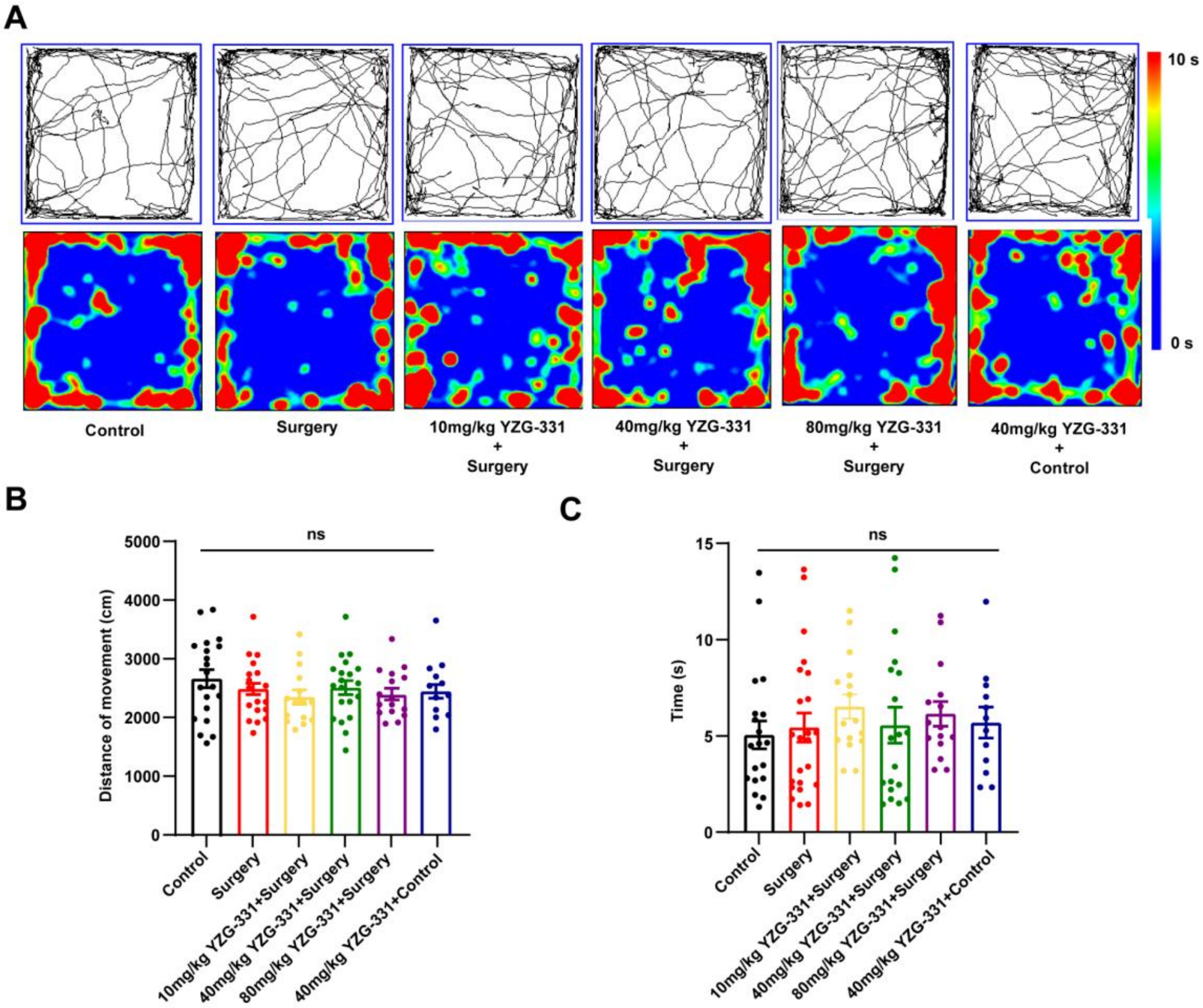

Figure S1 YZG-331 did not affect locomotor activity or anxiety-like behavior. (A) Representative movement tracks and time heatmap in the open field test. (B, C) Distance and duration of exercise in each group of mice (Control n = 20; Surgery n = 20; 10 mg/kg YZG-331+Surgery n = 15; 40 mg/kg YZG-331+Surgery n = 20; 80 mg/kg YZG-331+Surgery n = 15 and 40 mg/kg YZG-331+Control n =15). All data were analyzed using one-way ANOVA with Dunnett's test and are presented as mean ± SEM.

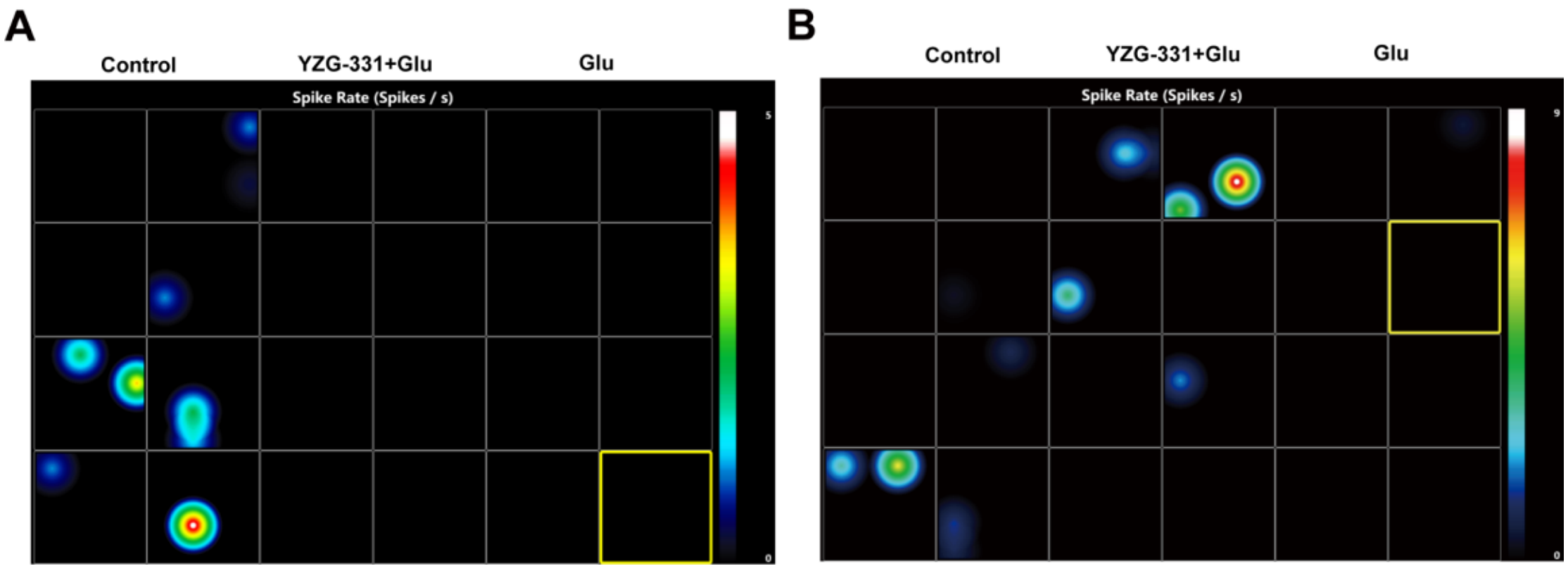

Figure S2 Network recording of neuronal electrical signals. (A) Recordings of neuronal electrical signal networks from three groups in the first hour of the testing period. (B) Recordings of neuronal electrical signal networks from three groups during the recovery phase.

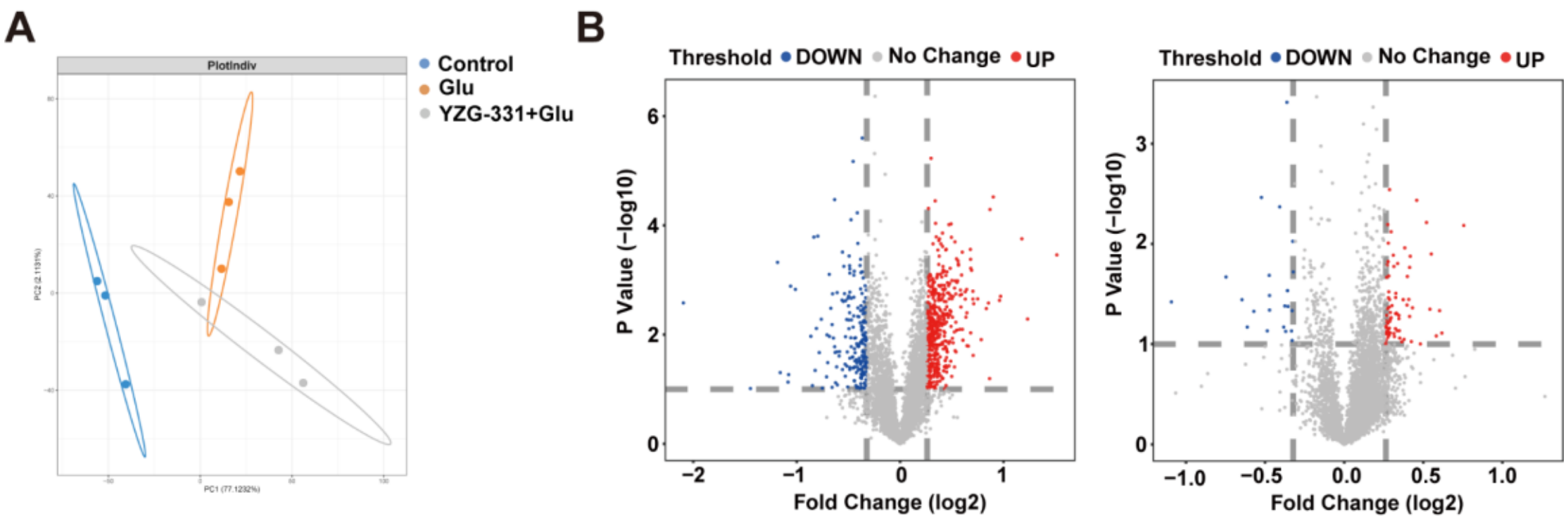

Figure S3 Multivariate and differential expression analysis of proteomic profiles. (A) Partial Least Squares Discriminant Analysis (PLS-DA) among the three groups. (B) Volcano plots of differentially expressed proteins (DEPs) between groups (left: Control *vs.* Glu; right: Glu *vs.* YZG-331 + Glu). DEPs; logFC ≥ 1.2 or ≤ 0.8; $^{*}P < 0.05$.

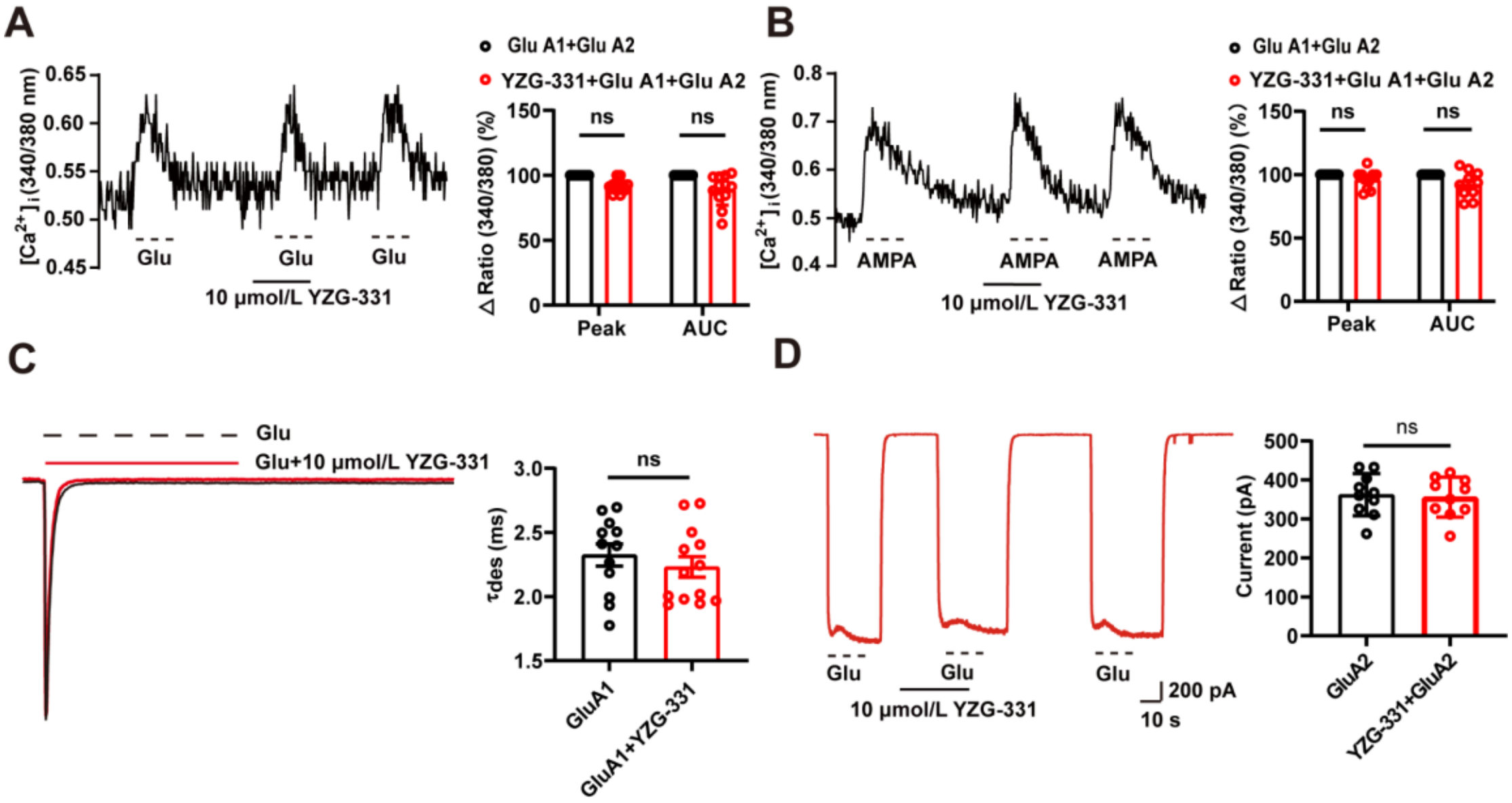

Figure S4 The effect of YZG-331 on AMPA receptor- and KA receptor-mediated electrical signals. (A, B) Representative traces illustrate the absence of inhibition of Glu A1/A2 and AMPA receptor-mediated elevations in $[Ca^{2+}]_i$ by YZG-331 in neurons. (C) Patch clamp was used to measure the degree of responsiveness of human Glu A1 to glutamate in neurons treated with or without YZG-331. (D) YZG-331 does not significantly alter the firing pattern of neurons in response to depolarizing currents.
